# Palmitic acid and palmitoylation in cancer: Understanding, insights, and challenges

**DOI:** 10.1016/j.xinn.2025.100918

**Published:** 2025-04-29

**Authors:** Peipei Song, Qiwei Jiang, Xueji Wu, Lang Bu, Wei Xie, Wenyi Wei, Xiaofang Xing, Jianping Guo

**Affiliations:** 1 Department of Laboratory Medicine, the First Affiliated Hospital, Sun Yat-sen University, Guangzhou 510275, China; 2 Institute of Precision Medicine, the First Affiliated Hospital, Sun Yat-sen University, Guangzhou 510275, China; 3 Department of Pathology, Beth Israel Deaconess Medical Center, Harvard Medical School, Boston, MA 02115, USA; 4 Department of Gastrointestinal Cancer Translational Research, Key Laboratory of Carcinogenesis and Translational Research (Ministry of Education), Peking University Cancer Hospital & Institute, Beijing 100191, China

**Keywords:** palmitic acid, palmitoylation, ZDHHC, metabolic homeostasis, tumor microenvironment

## Abstract

Nutrients from dietary foods not only provide energy and building blocks, but also play critical roles in modulating diverse pathophysiological functions. They achieve these, in part, by accelerating cell signaling transduction processes via modulating various types of protein post-translational modifications (PTMs). Notably, accumulating evidence has identified palmitic acid (PA), a major component of high-fat diets, as a significant contributor to various human disorders, including diabetes and cancer. Hence, further understanding the roles of PA and its involvement in protein palmitoylation, a key PTM, is crucial for uncovering the mechanisms underlying these diseases and exploring potential clinical applications in cancer therapy. This review comprehensively summarizes recent advances in the understanding of PA homeostasis and palmitoylation in tumorigenesis. Specifically, it highlights the connections between palmitoylation and key processes such as oncogenic signaling pathways, cell death mechanisms, innate immune responses, and the tumor microenvironment. The review also emphasizes potential therapeutic strategies, including targeting PA homeostasis, palmitoylation-associated processes, or specific palmitoylated proteins for cancer treatment. Finally, the challenges in the field, such as the regulation of PA homeostasis and the dynamic detection or targeting of palmitoylation, are discussed, underscoring the need for further research to address these critical issues.

## Public summary

• Palmitic acid (PA), primarily obtained from fatty and fried foods, plays a critical role in cancers.
• PA-induced palmitoylation regulates protein function and impacts various pathophysiological processes.
• Palmitoylated proteins are key modulators of inflammation and immune responses.
• Both PA and palmitoylation affect infection outcomes and the efficacy of tumor immunotherapies.

## Graphical abstract

**Figure:**
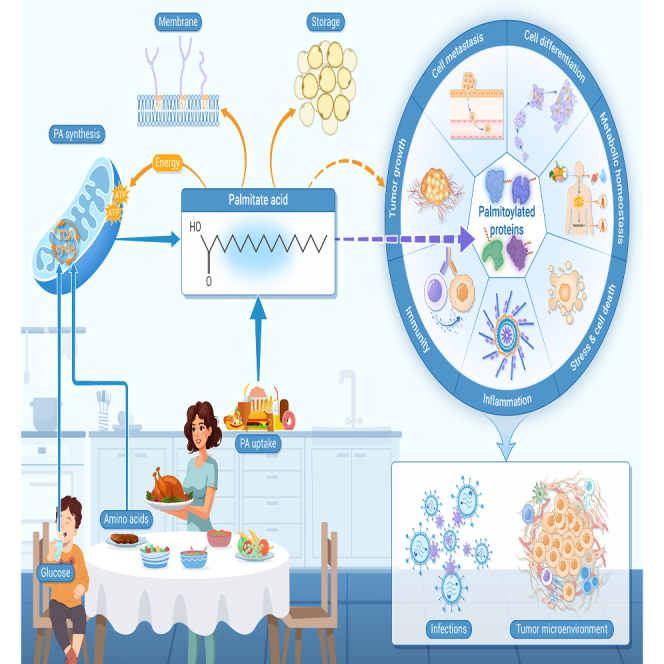

## Introduction

Metabolic homeostasis plays a pivotal role in maintaining the balance of life. Metabolic reprogramming, as a driver and adaptive response to intra- or extra-stimuli, has been elucidated as a hallmark of cancer, offering a balance of tumor autonomy or tumor microenvironments (TMEs) for tumor occurrence, progression, metastasis and drug resistance.^1^ On the one hand, alterations in a series of cellular metabolism pathways and energetics, including the production of energy units and building blocks by catabolic processes and anabolic processes involved in synthesizing complex biological molecules such as proteins, lipids, and DNA have been increasingly explored.^2^ On the other hand, certain intermediate metabolites play crucial roles in cell signaling transduction, acting as a cofactor or donor for protein post-translational modifications (PTMs), modulating multiple pathophysiological processes.

Specifically, lipid metabolism in tumor cells is usually elevated due to an increase in lipids required for plasma membrane (PM) synthesis and energy production for tumor cell growth and migration, as well as involvement in dynamic protein lipid modifications, enabling cells to integrate metabolic information into complex cellular decisions to ensure proper regulation of cellular processes. Among them, protein palmitoylation, as one of the most abundant and reversible PTMs, has been associated with regulating diverse protein functions, and is mechanistically linked to lipid metabolism and cellular energy homeostasis.

Here, we comprehensively review the metabolic homeostasis of lipids, in particular palmitic acid (PA), and extend its physiological function in protein palmitoylation in human diseases, especially in tumorigenesis and immune response. Understanding the specific mechanisms may provide new potential therapeutic targets or avenues for these disorders.

## High-fat diets and cancer

Diet ranks high on the list of important determinants of cancer risk in humans, not only increasing risk factors such as obesity and diabetes, but also affecting cell metabolism through a variety of mechanisms.^3^ Emerging reviews have highlighted their correlations as ubiquitous.^4^ In brief, three major nutrients, including glucose, lipids, and amino acids, are all tightly linked with tumorigenesis. For instance, glucose provides most of the energy for cancer cells to grow and proliferate; increased lipid metabolism in tumor cells promotes PM synthesis and energy generation; amino acids are central biomolecules of metabolism, creating a milieu that favors tumor initiation and progression, thereby playing controversial roles in tumorigenesis.

Of note, high-fat diets (HFDs) have been shown to be correlated with various tumors and accelerate the occurrence of cancer, attributed to inflammation and altered metabolism.^5^ Activation of lipid metabolism in tissues through excess fat consumption can favor tumor initiation, and a similar preconditioning could also occur in tumor metastasis.^6^ Exposure to HFDs can influence cancer development and progression. Mechanistically, HFDs promote colon tumor metastasis likely through increasing serum levels of cholesterol and cytokines and potentially inducing tumor angiogenesis.^7^ Another study found that HFDs promote peritoneal dissemination of gastric cancer,^8^ whereas treatment with a low-fat diet only inhibited lung metastasis of breast cancer.^9^ HFDs cause hyperlipidemia, which is closely related to the development of pancreatic cancer, and the inhibitory immune microenvironment is shaped by the reprogramming of cholesterol metabolism.^10^ Interestingly, chronic inflammation induced by HFD-induced obesity produces increased lung neutrophils and promotes lung metastasis in a mouse model of breast cancer, which can also be restored by weight loss.^11^ In mice, HFDs increase the intestinal progenitor stem cells (LGR5+) and then initiates tumors by activating the ubiquitous lipid-ligand transcription factor PPAR and ultimately enhancing fatty acid oxidation (FAO), which in turn regulates the storage and catabolism of dietary fats.^12^ Recent studies show that HFDs drive colorectal tumorigenesis mediated by gut microbial dysbiosis, metabolomic dysregulation, and gut barrier dysfunction in mice.^13^ It also shifts the gut microbiome and the breast TME to affect tumorigenesis.^14^

Importantly, with the increasing demand for vegetable oil worldwide, palm oil has become an important part of the global edible oil supply.^15^ PA is also more prevalent in many dietary fats, including fried food. Thus, PA functions involved in tumorigenesis and metastasis have been well established as a major source of lipids. To this end, emerging connection of PA-mediated protein palmitoylation and its connection with multiple pathophysiological functions have been elucidated and is summarized and discussed below.

## PA homeostasis and association with cancer

PA is a long-chain 16-carbon saturated fatty acid (FA), accounting for 44%–52% of total FAs of palm oil. Growing evidence suggests a strong link between PA diet and various diseases, including obesity,^16^ type 2 diabetes mellitus,^17^ cardiovascular diseases,^18^ and cancers.^15^^,^^19^ PA induces mitochondrial ROS generation and desensitizes the insulin signaling pathway via JNK (through TLR4) and impairs IRS-2 phosphorylation in hepatic cell lines; conversely, treatment with anti-oxidants attenuates the insulin resistance and reduces ROS generation.^20^ Recent studies have shown that a palm oil-rich diet fed to mice for 4 weeks led to changes in liver protein levels and S-palmitoylation with increased tumor risk factors.^21^ Besides, PA or HFDs specifically promote metastasis of oral cancer and melanoma in a CD36-dependent manner.^22^ Long-term adaptation to PA increases colorectal cancer (CRC) cell proliferation in a β-adrenergic receptor-dependent manner and promotes breast cancer formation.^23^^,^^24^

### Metabolic homeostasis of PA

**Figure 1:**
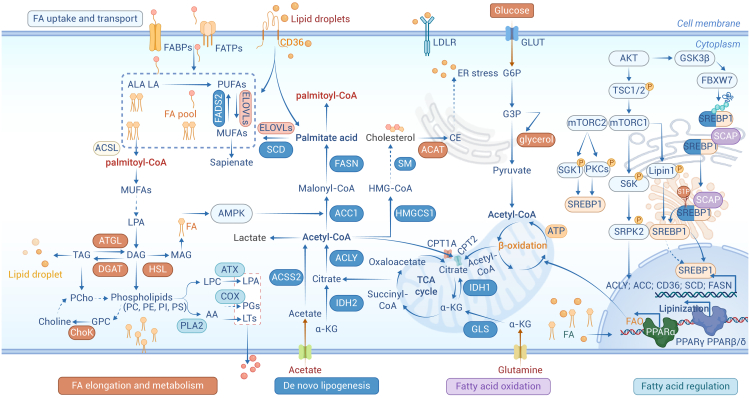
PA synthesis and metabolism Palmitic acid is mainly derived from the synthesis and catabolism of FAs. Dietary nutrients undergo the tricarboxylic acid cycle in the mitochondria to produce citric acid, which is transported to the cytoplasm through the citrate-pyruvate cycle to be converted to acetyl-CoA for *de novo* fat synthesis (FA and cholesterol). Acetate uptake via MCT is another source of acetyl-CoA in the cytoplasm. FASN condenses malonyl-CoA and acetyl-CoA to catalyze the production of saturated 16-carbon palmitate, which further elongates to MUFAs and PUFAs. Long-chain FAs are combined into triglycerides and stored in lipid droplets. Palmitic acid is converted to DAG, monoacylglycerol (MAG), and triglycerides. DAGs are also used in the synthesis of membrane phospholipids, and the major species include PC, PE, PI, and PS in the endoplasmic reticulum membrane, mitochondria, and cytoplasm. Lipids taken up by cells normally enter the intracellular pool of FAs, which are transported into mitochondria via the mitochondrial membrane protein CPT1A and undergo β-oxidation to produce acetyl-CoA. Glucose uptake by glucose transporters contributes to pyruvate and acetyl-CoA to support the tricarboxylic acid cycle in mitochondria. Lipid metabolic pathways cross each other to coordinate cellular metabolic states. Most of the enzymes involved in FA homeostasis are mainly regulated by sterol regulatory element-binding proteins (SREBPs). However, the active form of SREBP1 processed to mature is strongly influenced by PI3K-Akt-mTORC1-dependent mechanisms. Inhibition of mTORC1 resulted in suppression of the expression of key lipogenic enzymes including FASN, ACC1, and ACLY. On the one hand, mTORC1 directly phosphorylates LPIN1, which sequester mature SREBP1 to the nuclear periphery, resulting in inactivation by decreased activity and abundance of SREBP1 in the nucleus. On the other hand, mTORC1 promotes adipogenesis by activating S6K1 to phosphorylate SRPK2. mTORC2 can stimulate *de novo* lipogenesis through direct phosphorylation and activation of several SGK and PKC isoforms.

Overexpression of several lipase enzymes occurs early in tumorigenesis, which is observed in both proliferative and preinvasive stages. Thus, upregulation of *de novo* lipogenesis (DNL) may dynamically enhance oncogenic signals with each other throughout malignant transformation by regulating the enzymatic network of metabolism (Figure 1).^25^ PA is the most abundant saturated FA in the human body, serves critical roles in membrane structure and cellular signaling. While dietary intake constitutes a primary PA source, endogenous biosynthesis through DNL provides an alternative pathway, wherein metabolic precursors including carbohydrates, amino acids, and excess acetyl-CoA are enzymatically converted into FAs via coordinated actions of ATP-citrate lyase (ACLY), acetyl-CoA carboxylase (ACC), and fatty acid synthase (FASN).^26^ Cancer cells, in maintaining high DNL, have the flexibility to shunt them into different biosynthetic pathways.^27^ Both anabolic and catabolic pathways of FAs intersect, providing substrates for the synthesis of phospholipids and triacylglycerol (TAG) and fueling the cell through β-FAO in mitochondria. FA synthesis via DNL begins with the conversion of citrate to acetyl-CoA catalyzed by ACLY. Cytosolic citrate pools are supported through the TCA cycle or glutamine metabolism. As an alternative, acetate can escape from the requirement for citrate, directly contributing to acetyl-CoA via acetyl-CoA synthetases 2 (ACSS2).^28^ ACC catalyzes the generation of malonyl-CoA in the rate-limiting step of lipogenesis. Finally, the condensation of seven malonyl-CoA molecules and one acetyl-CoA by FASN catalyzes to produce the saturated 16-carbon palmitate, the main initial product of FA DNL (Figure 1).

Once generated or taken in, palmitate is then elongated by FA elongases (ELOVLs), or catalyzed at Δ9-desaturation by stearoyl-CoA desaturases (SCDs) to monounsaturated fatty acids (MUFAs) and saturated fatty acids (SFAs), governing the cellular MUFA/SFA ratio essential for membrane phospholipid biosynthesis, lipid droplet formation, triglycerides, wax esters, and cholesterol esters.^29^ Another FA desaturases (FADSs) can introduce double bonds at the Δ5 or Δ6 position, or Δ9 in long-chain FAs. Then synthesized PA is activated to palmitoyl-CoA by acetyl-CoA synthases (ACSLs). Fatty acyl-CoAs, including palmitoyl-CoA, are fused with glycerol and first converted into monoacylglycerols and followed by the addition of a second FA into diacylglycerols (DAGs). Diglyceride acyltransferase catalyzes the final step to produce TAG, which can then be sequestered and stored in low-density lipoproteins, synthesized within the endoplasmic reticulum (ER) membrane.

Dietary carbohydrates can provide a potential source of hepatic FA for DNL processes. Recently, protein has been validated as the major source of carbon to promote hepatic steatosis,^30^ indicating the harmful role of a high protein diet in MASLD and arteriosclerosis.^31^ Since endogenous biosynthesis balances external intake, the distribution and metabolism of PA do not significantly influence this homeostatic rigorous static control, which may be related to its basic physiological role in guaranteeing the new physical energy of biofilms. However, the special physiological environment may lead to the increase of PA by affecting DNL. There is substantial evidence that DNL is significantly induced in diseases such as obesity, insulin resistance, and steatosis, greatly promoting changes in liver fat deposition and FA composition.^32^

### Key enzymes in PA metabolism: SCD and CDP-DAG synthase

In oncogenic contexts, the SCD enzymes are crucial for tumorigenesis since they regulate the pool of cellular unsaturated FA that serve as building blocks for various lipids. SCD is associated with cancer cell proliferation and survival, which may be a potential link between metabolic disorders and cancer development.^29^ Mechanistic studies suggest that this linkage may occur by sustaining proliferative signaling through the assembly of MUFA-enriched membrane microdomains, which enhance the activation of growth factor receptor tyrosine kinases such as EGFR and HER2.^33^ Additionally, SCD enzymes promote resistance to apoptosis by producing oleate-rich lipid species that inhibit the formation of mitochondrial permeability transition pores.^34^ Overexpression of SCD1 attenuated PA-induced podocyte death.^35^ Studies revealed that SCD was overexpressed in tumors in prostate, liver, and kidney cancer,^36^^,^^37^^,^^38^ and it also correlates with shorter survival in breast cancer.^39^ However, silencing of *SCD1* and several lipid synthesis enzymes reduced the viability of breast cancer cell lines.^40^ Consistently, treatment with SCD inhibitors reduced tumor formation in xenograft models of gastric and colon cancer.^41^ Clinical correlative analyses have demonstrated positive associations between SCD1 overexpression and adiposity indices (BMI), markers of insulin resistance, and characteristics of the metabolic syndrome phenotype. Knockout of *Scd1* decreases endogenous monounsaturated FAs and body adiposity, increases insulin sensitivity, and protects against diet-induced obesity.^42^ Notably, SCD1-mediated lipid desaturation activates pro-tumorigenic pathways including PI3K/AKT/mTOR and Wnt/β-catenin signaling, while simultaneously suppressing AMPK-dependent metabolic surveillance. This pleiotropic regulation positions SCD1 as a molecular nexus bridging metabolic dysfunction and oncogenic transformation, particularly in obesity-associated malignancies.^37^

Recent evidence suggests that, besides SCD enzymes, FADS2 also plays a role in FA desaturation, and cancer cells utilize a FADS2-dependent alternative pathway to transfer palmitate to sapienate to support cell proliferation.^43^ This alternative metabolic pathway ensures the production of MUFAs when SCD is inhibited, thereby offering the strategy of combined inhibition of both FADS2 and SCD to reduce hepatocellular carcinoma.^43^

Oleic acid (C18:1ω9) is a type of unsaturated FA derived from palmitate, which can be fed into phosphatidic acid synthesis by incorporating with TAG for storage. Importantly, phosphatidic acid can be catalyzed by CDP-DAG synthase to generate CDP-DAG, a main precursor for the *de novo* synthesis of complex glycolipids such as phosphatidylserines (PSs), phosphatidylglycerols (PGs) and phosphatidylcholines (PCs), which support cellular proliferation and survival in tumorigenesis.^44^ Omega-3 FAs eicosapentaenoic acid (EPA), docosahexaenoic acid (DHA) and omega-6 arachidonic acid (AA) are essential polyunsaturated fatty acids (PUFAs). Unless directly dietary ingested, they must be desaturated and elongated, derived from the short-chain omega-3 α-linolenic acid and the omega-6 linoleic acid, respectively.^45^ EPA and DHA generally reduce inflammatory processes and are believed to lower the risk of breast and other cancers.^46^ AA is also produced from membrane phospholipids in response to phospholipase A2 and released into the cytoplasm, which is the substrate for the synthesis of eicosanoids (prostaglandin and thromboxane) via the cyclooxygenase pathway and leukotrienes via the lipo-oxygenase route, these molecules mediate pro-inflammatory and pro-tumorigenic pathways (Figure 1).^47^

### Key proteins for PA uptake and transport: CD36, FATPs, FABPs

Exogenous uptake of FA appears to be a general mechanism of cancer cell dissemination and resistance to therapy. Specialized transporters are required for FA uptake to facilitate efficient movement across the PM, including CD36 and fatty acid transport proteins (FATPs). CD36, also known as FA translocase, is a scavenger receptor expressed in multiple cell types, mediating lipid uptake, immunological recognition, apoptosis, and other biological processes.^48^ Cancer cells expressing high levels of the receptor CD36 and lipid metabolism genes are more likely to initiate metastasis.^22^ In metastasis-initiating cells, CD36 primarily functions to internalize long-chain fatty acids (LCFAs) that enhance cellular metastatic potential.^22^ In the case of metabolic abnormalities, CD36-mediated inhibition of AMPK activation may promote DNL and cancer cell proliferation.^49^ CD36 binds to extracellular ligands oxLDL and facilitates cellular LCFA uptake across the PM.^50^ Following cellular uptake through CD36, LCFAs are converted to palmitoyl-CoA. LCFAs are transported into the cell and then into the mitochondria matrix through a series of transport machinery interactions including FABP4, ACSL1, CPT1, and CPT2 (Figure 1). The binding, accompanied by CD36 cytosolic tail, recruits a signalosome complex, including SFKs, JNK1/2, and Vav, which is also enhanced by NOX-derived ROS.^51^ Expression of CD36 is regulated at both the transcriptional and post-translational levels. ZDHHC4 palmitoylates newly synthesized CD36 in the Golgi and vesicle-mediating trafficking it into the PM, while ZDHHC5 could maintain the PM localization of CD36 by preventing the depalmitoylation of CD36.^52^ Another study showed that, during the process of FA uptake, CD36 was under the control of dynamic palmitoylation regulation. The downstream kinase LYN phosphorylates ZDHHC5 and inactivates it, and CD36 is further depalmitoylated by acyl protein thioesterase 1 (APT1) to initiate endocytic uptake of FA.^53^ Palmitoylation of CD36 enhances FA uptake and CD36/Fyn/Lyn complex formation, thereby promoting the development of liver cancer.^53^ In addition, the PPAR-γ pathway also upregulates genes involved in intracellular LCFA transport, including FABP4.^54^

FATPs are a group of integral membrane proteins, also known as solute carrier protein family 27 (SLC27), expressed on the tumor cells’ surface to increase intracellular lipid availability and support cancer progression and therapy resistance.^55^ FATPs consist of six isoforms that are tissue-specific and involved in the progression of several cancers, including breast, prostate, liver, and lung cancer. Furthermore, FATPs can function as direct transporters of LCFA and as bifunctional proteins with independent transport and enzymatic activity for its translocation from the cytoplasm to the PM.^56^

The regulation of exogenous lipid uptake primarily depends on the overexpression of HIF-dependent lipid-binding proteins under hypoxia. FABP4 binds with PA and produces inflammatory responses such as chemokine- and TNF-α-NF-κB signaling pathways under obesity-induced stress conditions.^57^ The higher circulatory FABP4 facilitates cancer cell metabolism associated with lipid metabolism, promoting the growth, progression, and survival of PCa cells.^58^ In addition, FABP4 increases the metastatic potential of ovarian cancer by regulating pathways related to ovarian cancer cell metabolism.^59^ FABP4 also regulates angiogenic and metabolic signaling in tumor-associated endothelial cells, in which the transcription factor FOXO1 promotes VEGFA-induced FABP4 expression.^60^ On the other hand, FABP5 promotes cancer invasion by inducing the epithelial-to-mesenchymal (EMT) transition of hepatocellular carcinoma (HCC), and targeting FABP5 results in altered profiles of lipid signaling intermediates and decreases lipid-mediated invasion in PCa cells.^61^ Moreover, FABP3 and FABP7 are involved in FA uptake, knockdown of them reduced FA uptake, and inhibition of lipid droplet (LD) formation under hypoxic conditions, thereby decreasing the growth of U87 glioblastoma tumors.^62^

### Enzymes in PA synthesis and regulation: ACLY, ACC, ACSS, FASN, SREBP

ACLY is a key enzyme that directs excess glycolytic flux ATP to lipid synthesis to promote tumor growth and differentiation. As such, ACLY is upregulated in a variety of tumors, and inhibition of ACLY genetically or pharmacologically significantly inhibits tumor growth in lung, prostate, or ovarian cancer xenograft mice.^63^ It has been reported that AKT directly phosphorylates and enhances ACLY activity, leading to the increase of acetyl-CoA synthesis,^64^ while the acetylation of ACLY blocks its ubiquitylation and degradation.^65^

The conversion of acetyl-CoA to malonyl-CoA is an important step for FA synthesis, which is mediated by ACC. ACC is upregulated in head and neck squamous cell carcinoma and glioblastoma.^66^^,^^67^ Inhibition of ACC significantly reduces FA synthesis and suppresses tumor growth in various xenograft models.^68^ ACC is mainly phosphorylated and inhibited by the energy sensor kinase AMPK, reducing the generation of malonyl-CoA and stimulating fat β-oxidation.^69^ Recent studies also indicate the hydroxylation of ACC2 by Egln2 in the liver and muscles, involving in FAO repression, which is independent of HIF- and AMPK-mediated phosphorylation (Figure 1).^70^

ACSSs convert acetate to acetyl-CoA, making acetate an important molecule for lipid synthesis and histone acetylation. Especially, cancer cells rely on acetate as a carbon source for FA synthesis under low-oxygen and lipid-depleted conditions.^28^ One study showed that adult mice with *Acss2* deficiency exhibit a significant reduction in tumor burden in hepatocellular carcinoma without deficits in growth or development.^71^ In addition, ACSS2 expression correlates inversely with overall survival in patients. Oncogenic *KRAS* mutations synergize with ACLY and ACSS2 enzymatic activities to drive tumor cell proliferation and metastatic progression.^72^ Furthermore, one recent study found that O-linked *N*-acetylglucosamine transferase, a glycosylation enzyme increased in many tumors and promoted tumor growth, reduce its polyubiquitination and degradation by phosphorylation of ACSS2 in a cyclin-dependent kinase 5 (CDK5)-dependent manner, resulting in subsequent stimulation of acetate-driven DNL.^73^

FASN is a key enzyme with multifaceted roles in supporting both anabolic metabolism and oncogenic signaling. Its inactivation has been shown to impair hepatocarcinogenesis driven by AKT in mice and humans.^74^ However, cancer cells could adapt to FASN inhibition by switching from *de novo* FA synthesis to FA uptake. This adaptive mechanism may even promote metastasis due to the induction of EMT.^75^ Currently, the FDA has approved drugs targeting FASN for obesity treatment, which also display potential role in blocking lipid synthesis in tumors; however, the clinical application of these drugs is limited by the development of drug resistance.^76^

The regulation of most enzymes involved in FA synthesis occurs largely at the transcriptional level through the activation of sterol regulatory element-binding proteins (SREBPs). There are three main SREBP isoforms: SREBP1a and SREBP1c, which arise from alternative splicing of *SREBPF1* gene, and SREBP2 encoded by the *SREBPF2* gene.^77^ While SREBP1c primarily regulates FA biosynthesis and SREBP2 preferentially regulates cholesterol biosynthesis, all isoforms have the ability to activate genes within both pathways.^77^^,^^78^ The processing of SREBP1 into its mature, active form is strongly influenced by PI3K-AKT-mTORC1-dependent mechanisms.^79^ Additionally, PTEN targets PIP3 to block AKT activation, thereby inhibiting FASN expression. Key lipogenic enzymes, including FASN, ACC1, and ACLY, are suppressed following mTORC1 inhibition by rapamycin inhibition or *Raptor* knockdown.^80^ mTORC1 directly phosphorylates the phosphatidic acid phosphatase lipin1 (LPIN1), which inactivates mature SREBP by sequestering it at the nuclear periphery, leading to decreased activity and abundance of the nuclear SREBP.^81^ Another mechanism involves mTORC1 promoting lipogenesis via SRPK2, a key regulator of RNA-binding proteins. mTORC1 activated S6 kinase 1 (S6K1) phosphorylates SRPK2, promoting its nuclear translocation and phosphorylation of SR proteins. SRPK2 promotes SR protein binding to U1-70K, increasing mRNA splicing of genes involved in DNL.^82^ On the other hand, mTORC2 promotes cancer by facilitating the formation of lipids essential for growth and energy production. It stimulates DNL by directly phosphorylating and activating several isoforms of SGK and PKC (Figure 1).^83^

### Enzymes in FAO: CPT1A, CPT2

Carnitine palmitoyl transferase 1A (CPT1A) is a central regulatory enzyme in the FAO pathway, catalyzing the transport of FAs into the mitochondria, thereby promoting their oxidation to produce energy. Furthermore, CPT1A also enhances cellular pluripotency and metabolic transitions through lipid remodeling and metabolic reprogramming, which are crucial for cell fate determination.^84^ Increased expression of CPT1A in radiation-resistant nasopharyngeal carcinoma (NPC) cells is significantly associated with poor overall survival of NPC patients.^85^ However, emerging evidence indicates that HCC specimens harboring TP53 mutations exhibit compromised CPT1A functionality, which induces metabolic dysregulation characterized by intracellular accumulation of branched-chain amino acids. This aberrant metabolite accumulation subsequently drives constitutive activation of the mTOR signaling cascade, establishing a protumorigenic microenvironment that potentiates hepatic oncogenesis through enhanced proliferative signaling and metabolic reprogramming.^86^ Although there are no current reports on the palmitoylation of CPT1A, it can serve as a stabilizer for MAVS activation, recruiting the ER-localized ZDHHC4 to catalyze the palmitoylation of MAVS. This promotes the stabilization and activation of MAVS, enhancing the IFN-I response and strengthening antiviral defenses and antitumor immune therapy.^87^

CPT2, located on the inner of mitochondrial membrane, catalyzes the conversion of acylcarnitine back to CoA in the intermembrane space, making it available for β-oxidation. CPT2 plays contrasting roles in different tumors. Its expression is significantly increased in recurrent breast cancer and chronic lymphocytic leukemia, but frequently downregulated in CRC, HCC, and primary ovarian serous carcinomas. Low CPT2 expression is an independent prognostic factor for poorer overall survival.^88^ Downregulation of CPT2 could inhibit p53 expression, activate the ROS pathway, or enhance SCD1-mediated FA biosynthesis, thereby promoting tumor cell proliferation and metastasis.^89^^,^^90^ However, the role of CPT2 in palmitoylation remains to be further explored. We summarized the genetic mouse models for PA homeostasis-associated proteins as below (Table 1).

**Table 1:** Genetic mouse models for PA homeostasis-associated proteins

Tissue specificity	Genes	Manifestations	References
Whole body	*Cd36*^−/−^	less lipid droplets and inflammatory cell infiltration, decreased content of triglyceride	Zhao et al.^91^
Liver	*Cd36*^−/−^	attenuated HFD-induced fatty liver due to decreased hepatic FA uptake	Wilson et al.^92^
Liver	*Scd*^−/−^	lipogenesis and levels of nuclear SREBP-1 and CHREBP were significantly decreased	Miyazaki et al.^93^
Whole body	*Scd*^−/−^	reduced body adiposity, increased insulin sensitivity, and resistance to diet-induced weight gain	Ntambi et al.^42^
Pancreas	*Acly^-^*^*/-*^	not cause overt metabolic abnormalities, but acinar-to-ductal metaplasia and reduced pancreatic tumor formation	Carrer et al.^94^
Fat tissue	*Acly^-^*^*/-*^	impaired thermogenesis, reduced expression of UCP1 and other thermogenic genes, overload of the TCA cycle	Korobkina et al.^95^
Liver	*Acss2*^−/−^	reduced in body weight and tumor burden in a diet-induced obesity model	Huang et al.^71^
Whole body	*Acss2*^−/−^	reduced the absolute number and inhibited tumor growth of liver tumors	Comerford et al.^72^
Liver	*Fasn*^−/−^	abolished lipid accumulation in mouse hepatocytes, inhibition of hepatocarcinogenesis in AKT overexpressing	Li et al.^74^
Adipocyte	*Fabp4*^−/−^	insulin-sensitive, lower plasma triglyceride and cholesterol levels	Uysal et al.^96^
Adipocyte	*Fabp4*^−/−^	reduced expression of inflammatory cytokines in macrophages	Furuhashi et al.^97^
Macrophages	*Fabp4*^−/−^	enhanced insulin signaling and glucose uptake in adipocytes	Furuhashi et al.^97^
Whole body	*Fabp4*^−/−^*;* *Fabp5*^−/−^	enhanced insulin receptor signaling and AMPK activity, and reduced liver SCD-1 activity	Maeda et al.^98^
Whole body	*Fabp4*^−/−^	insulin resistance, increasing lipid availability and supporting metastasis	Nieman et al.^99^
Whole body	*Fatp4*^−/−^	displayed epidermal hyperplasia and hyperkeratosis	Lin et al.^100^
Adipocytes	*Fatp4*^−/−^	increased weight gain, visceral adipocyte hypertrophy, and hepatic steatosis on high fat or LCFA diet, no changes in insulin sensitivity and adipose inflammation	Lenz et al.^101^
Whole body	*Fatp1*^−/−^	reduced skeletal muscle lipid accumulation; improved insulin sensitivity after lipid challenges	Kim et al.^102^
Whole body	*Fatp1*^−/−^	abolished insulin-stimulated LCFA uptake by muscle and adipose tissue; redistribution of lipids from fat and muscle to liver	Wu et al.^103^
Liver	*Fatp2*^−/−^	protection from hepatosteatosis and improved glucose levels and insulin sensitivity following high-fat diet	Falcon et al.^104^
Whole body	*Fatp4*^−/−^	features of lethal restrictive dermopathy	Moulson et al.^105^
Whole body	*Fatp5*^−/−^	enlarged hepatocytes; reduced LCFA uptake in hepatocytes and triglyceride levels in liver; increased FA synthetase expression and *de novo* FA synthesis	Doege et al.^106^
Liver	*Fatp5*^−/−^	reduced FA absorption in liver and increased lipid deposition in heart, muscle, and fat; reversal of hepatic steatosis; improved whole-body energy homeostasis	Doege et al.^107^
Liver	*Cpt1a*^−/−^	increased tumor numbers and larger surface tumor sizes, with promoted lipid droplets accumulation	Liu et al.^86^
Adipose-specific	*Cpt2*^−/−^	failed to induce the expression of thermogenic genes in response to adrenergic stimulation	Lee et al.^108^

## Protein palmitoylation and their roles in cancer

Similar to other PTMs, palmitoylation involves the covalent attachment of palmitoyl-CoA to cysteine residues via a reversible thioester linkage, catalyzed by the zinc finger DHHC-type containing (ZDHHC) protein family. The biological significance of protein palmitoylation was not fully recognized until 2002, with the identification of yeast protein acyltransferases (PATs), enzymes that specifically catalyze the addition of palmitoyl groups to cysteine.^109^ The reversibility of palmitoylation makes it a unique and dynamic mechanism for regulating protein activity, stability, subcellular localization, membrane shuttling, protein-protein interactions, enzymatic activity, and various other cellular processes.^110^

### Enzymes for catalyzing palmitoylation: ZDHHC protein family

Protein palmitoylation typically involves the attachment of a 16-carbon palmitate to substrate proteins. However, a subset of proteins can be modified by fatty acyl chains of varying lengths.^111^ Palmitoylation is mediated by a protein family sharing a conserved Asp-His-His-Cys (DHHC) motif within a cysteine-rich domain. During palmitoylation, the DHHC domain of ZDHHC binds to palmitoyl-CoA located in the membrane, undergoes automatic palmitoylation, and subsequently transfers the palmitate group to cysteine residues on the substrate protein, facilitating its membrane localization.^112^ To date, 23 palmitoyl transferases have been identified in human protein databases, designated from DHHC1 to DHHC24 (excluding DHHC10). These enzymes regulate a large number of proteins undergoing palmitoylation.^113^

**Figure 2:**
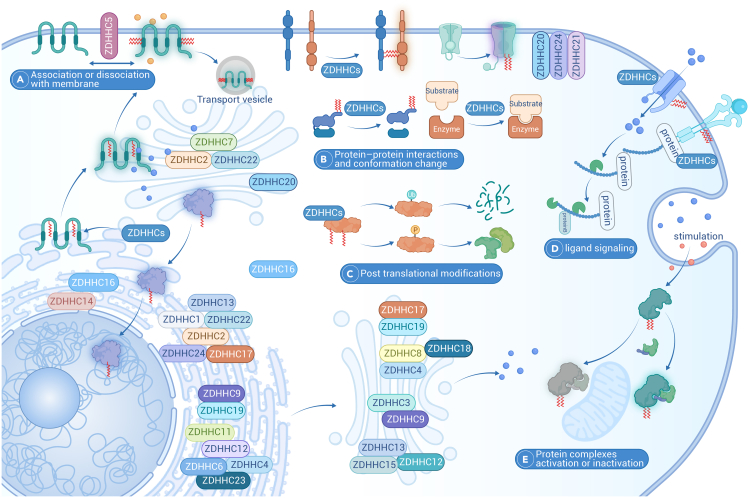
Location and function of ZDHHCs There are 23 palmitoyl transferases known in human protein databases, designated from ZDHHC1 to ZDHHC24 skipping ZDHHC10. Plasma membrane-localized ZDHHCs (ZDHHC5, 20, 21, 24) promote local palmitoylation and drive their localization, many target at the endoplasmic reticulum (ZDHHC1, 2, 4, 6, 9, 11, 12, 13, 17, 19, 22, 23, 24) and some localize at Golgi (ZDHHC2, 3, 4, 7, 8, 9, 12, 13,15, 17, 18) mediate the palmitoylation of their substrates and location. While some numbers, such as ZDHHC2, 4, 12, 13, 16, 19, 22, appear at several different membrane locations. The membrane localization of ZDHHCs is not stationary, they might be flexible, dynamically shuttling among inner membrane for their functions. The most fundamental effect of ZDHHCs on substrate palmitoylation is the alteration of subcellular localization, which run through the protein synthesis, transportation, and membrane fusion (A). Another important aspect is to regulate protein interaction and conformation thereby activating enzyme activity or protein complex function (B). Palmitoylation also regulates protein stability and degradation by post-translational modification, particularly, ubiquitination and phosphorylation (C). Palmitoylation can also act as a cellular signaling switch that activates signaling by altering membrane receptors or ligands to recruit downstream signaling molecules (D and E).

ZDHHCs are integral membrane proteins with at least four transmembrane helices, with the catalytic domain located between them. Structural studies indicate that the conserved Thr-Thr-X-Glu (TTXE) and Asp-Pro-Gly (DPG) domains are located in the cytoplasmic region, with C- and N-terminal cytoplasmic tails mediating protein-protein interactions, thereby contributing to the acyl transfer process.^114^ The two Zn^2+^ ions also play a role in stabilizing structural and are positioned between three parallel layers of β hairpins. The membrane localization of ZDHHCs is flexible, and different splicing isoforms may exhibit distinct localization, biological function, catalytic efficiency, and donor preferences (Figure 2).^114^

Some ZDHHCs primarily localize to the PM (e.g., ZDHHC5, ZDHHC20, ZDHHC23), promoting local palmitoylation and driving the localization of their substrates. Others target the ER (e.g., ZDHHC1, ZDHHC6, ZDHHC11, ZDHHC24) and the Golgi (e.g., ZDHHC3, ZDHHC7, ZDHHC15, ZDHHC18), mediating the palmitoylation of their substrates and influencing their localization. The precise selection of substrate protein for palmitoyltransferase remains incompletely understood. However, it has been observed that ZDHHC13 and ZDHHC17 possess distinctive C-terminal ankyrin-repeat domains that interact with specific proteins, promoting their membrane localization, and facilitating palmitoylation by other ZDHHC enzymes.^115^ Thus, co-localization of ZDHHC with its substrate is critical for driving the correct palmitoylation event.^116^ Meanwhile, the same substrate (at different sites) may be catalyzed by distinct ZDHHCs. For example, IFN-induced transmembrane (IFITM3), a cellular endosome and lysosome localized protein that restricts numerous virus infections, can be palmitoylated by more than half of the ZDHHCs, including ZDHHC3, ZDHHC7, ZDHHC15, and ZDHHC20, ensuring a robust antiviral response.^117^ The potential roles of these ZDHHCs in tumorigenesis are summarized below (Figure 2; Table S1).

#### Tumor suppressor-like ZDHHCs: ZDHHC1, 2, 7, 13, 22

ZDHHC1 has been identified as a potential tumor suppressor, with its expression frequently silenced in multiple tumor cells due to promoter methylation. Restoration of ZDHHC1 expression can inhibit cancer cell progression by stimulating apoptosis, inducing cell-cycle arrest, and repressing metastasis.^118^ ZDHHC1 upregulates several target genes involved in ER stress and pyroptosis, including NLRP3, caspase-1, IL-1β, and IL-18. It also negatively regulates tumor cell metabolism, offering potential targets for anticancer therapy.^119^ Overexpression of ZDHHC1 suppresses prostate cancer cell tumorigenicity and increases the palmitoylation of IGF2BP1,^120^ reducing the stability of LIPG in an m6A-dependent manner.^121^ ZDHHC1 is also a p53-dependent tumor suppressor that mediates the palmitoylation of p53, promoting its translocation from the cytosol to the nucleus and activating p53 signaling. Moreover, ZDHHC1 expression is suppressed in most p53-wild-type (p53-WT) cancer cells due to the recruitment of DNMT3A by p53-WT, leading to hypermethylation and subsequent inactivation and degradation of non-palmitoylated p53, thereby promoting tumorigenesis.^118^

ZDHHC2 is a palmitoyl transferase specific for CD9 and CD151, protecting them from lysosomal degradation. ZDHHC2 functions as a tumor suppressor, and its reduced expression predicts poor prognosis in gastric adenocarcinoma patients and is associated with lymph node metastasis. ZDHHC2 mediates AGK palmitoylation, promoting the translocation of AGK into the PM and activation of the PI3K-AKT-mTOR signaling pathway in clear cell renal cell carcinoma.^122^ Palmitoylation of JAM-C by ZDHHC7 promotes its localization to tight junctions and inhibits migration of A549 lung cancer cells.^123^ ZDHHC7 also mediates palmitoylation of the tumor suppressor protein SCRIB, and knockdown of *ZDHHC7* results in its translocation, YAP activation, and loss of inhibitory activity against oncogenic pathways.^124^ ZDHHC13 palmitoylates MC1R, suppressing UVB-induced transformation of human melanocytes and delaying melanomagenesis, a process further enhanced by AMPK-mediated phosphorylation of ZDHHC13.^125^ Another study showed that the enzymatic activity of ZDHHC13, rather than protein scaffolding function, is essential for skin barrier integrity.^126^ ZDHHC22 has been shown as a tumor suppressor due to reducing mTOR stability via palmitoylation, decreasing AKT signaling pathway activation, and restraining breast cancer growth.^127^

#### Oncogene-like ZDHHCs: ZDHHC3, 4, 5, 6, 8, 9, 11, 12, 15, 17, 18, 19, 23, 24

*ZDHHC3* ablation, in combination with chemotherapy, enhances anti-cancer cell activities, not only by increasing oxidative stress but also by enhancing the anti-growth effects of chemotherapeutic agents.^128^ ZDHHC3-mediated protein palmitoylation supports breast tumor growth by modulating cellular oxidative stress and senescence.^129^ Additionally, ZDHHC3 palmitoylates ITGA6 and ITGβ4, the activation of which increases cell migration.^130^

Depletion of either *ZDHHC4* or *ZDHHC5* in cells disrupts CD36-dependent FA uptake. Adipose-specific *Zdhhc5* knockout mice show decreased FA uptake activity in adipose tissues.^52^ ZDHHC4 mediates the palmitoylation and activation of GSK3β, sustaining the stemness of temozolomide-resistant glioblastoma multiforme (GBM) by activating the EZH2-STAT3 signaling axis.^131^ ZDHHC5, primarily located at the PM, is involved in the regulation of pattern recognition receptor signaling and protein localization to the PM.^132^ It is indispensable for the palmitoylation and membrane localization of adhesion proteins.^133^ ZDHHC5 contributes to the self-renewal of glioma-initiating stem cells through the palmitoylation of EZH2, activating histone H3 Lysine 27 trimethylation (H3K27me3), and driving malignant development and progression in *p53*-mutant gliomas.^134^ Additionally, ZDHHC5 has been identified as capable of palmitoylating cardiac fibroblast cell membrane proteins, triggering massive endocytosis and regulating the cardiac sodium pump.^135^^,^^136^

ZDHHC6 is a downstream substrate for DHHC16, and its deletion enhances tumor progression in a DEN-induced HCC mouse model by suppressing AEG-1 palmitoylation.^137^ *ZDHHC6* knockdown reduces MYD88 palmitoylation and TLR/MYD88 signaling upon activation, making it a potential therapeutic target for sepsis.^138^ *ZDHHC8* depletion enhances radiosensitivity and suppresses tumor growth in a mesothelioma mouse model.^139^ ZDHHC8 is responsible for palmitoylating ABCA1 and D2 dopamine receptor, regulating their localization to the PM.^140^ ZDHHC9 plays a key role in brain development and behavior, and its palmitoylation of GLUT1 promotes glioblastoma glycolysis and tumorigenesis,^141^ while stabilizing PD-L1 to promote breast tumor growth.^142^

ZDHHC11 acts as a key oncogene in maintaining high MYB levels induced by MYC in Burkitt lymphoma.^143^ It also modulates the activity of MITA in the DNA virus-triggered innate immune response, facilitating the optimal recruitment of IRF3 to MITA.^144^ Recent research indicates that ZDHHC11 modulates LD catabolism by palmitoylating adipose triacylglycerol lipase (ATGL). Inhibition of ATGL palmitoylation renders it catalytically inactive, even when properly localized, causing LD accumulation, defects in lipolysis, and impaired lipophagy.^145^ ZDHHC12-mediated CLDN3 palmitoylation promotes accurate membrane localization, protein stability, and ovarian cancer cell tumorigenesis.^146^ Notably, ZDHHC12 acts as a repressor of NLRP3 inflammasome activation, enhancing NLRP3 degradation through the chaperone-mediated autophagy pathway.^147^ Mutations in *ZDHHC15* are mostly associated with neuropsychiatric diseases, and its palmitoylation regulates dopamine signaling in the striatum.^148^ Moreover, ZDHHC15 expression is significantly upregulated in glioma, promoting malignancy via the STAT3 signaling pathway, offering a novel prognostic biomarker for glioma patients.^149^

A large number of studies demonstrated that ZDHHC17 was involved in axon growth and the palmitoylation of synapsis-associated proteins. It recognizes substrates through ZDHHC ANK binding motif (zDABM)-dependent or zDABM-independent mechanisms, with some substrates displaying multiple binding modes.^150^ ZDHHC17 drives GBM development and malignant progression via the MAP2K4-JNK/p38 signaling module.^151^ AMPKα is also palmitoylated by ZDHHC17, which is required for AMPK activation and subcellular membrane compartmentalization.^152^ Another study found that OCT4A was a substrate of ZDHHC17, and its palmitoylation is indispensable for preserving it from lysosome degradation and enhancing stemness in glioblastoma.^153^ Both ZDHHC17 and ZDHHC24 play an oncogenic role in HCC by palmitoylating AKT at different residues both in cells and knockout mouse models, a process likely necessary for HFD-induced MASH and HCC.^154^

ZDHHC18-mediated palmitoylation of MDH2 maintains mitochondrial respiration and enhances ovarian cancer cell growth and colony formation.^155^ ZDHHC18 and ZDHHC23 interact with RNF144A, ZDHHC23 overexpression downregulates BMI1 protein level by increasing polyubiquitination, while ZDHHC18 has the opposite effect. Additionally, high expression of ZDHHC18 relates to the mesenchymal molecular phenotype and that of ZDHHC23 is related to the proneural phenotype in glioblastomas, respectively. This supports the role of ZDHHC18 in promoting the survival of mesenchymal GBM contain stem cell-like tumor cells (GSCs) under stress microenvironment by increasing BMI1 protein stability.^156^ It was reported that ZDHHC19 accelerates tumor progression through the Wnt/β-catenin pathway in osteosarcoma.^157^ Recent study showed that Flotillin-1 is catalyzed by ZDHHC19 and APT1 for the turnover palmitoylation, preventing degradation of IGF-1R via endocytosis and lysosomal pathways, thereby inducing EMT, migration, and invasion of cervical cancer.^158^

#### Context-dependent function of ZDHHCs: ZDHHC7, 14, 16, 20, 21

Except as a suppressor, ZDHHC7 has been reported to have oncogenic functions by mediating the palmitoylation of STAT3, resulting in elevated HIF1A levels. CDK5 stabilizes HIF1α, enabling it to promote ZDHHC7 expression, creating a positive feedback loop between ZDHHC7, STAT3, and HIF1α.^159^ ZDHHC14 overexpression is associated with acute biphenotypic leukemia and increased invasiveness of gastric cancer cells,^160^^,^^161^ but it has also been identified as a novel human tumor suppressor gene.^162^ ZDHHC14 regulates G protein-coupled receptor (GPCR) signaling by palmitoylating the β2-adrenergic receptor, trafficking it to the Golgi complex for palmitoylation and then to the PM for depalmitoylation.^163^ Additionally, ZDHHC14 interacts with and palmitoylates PSD93 and Kv1 potassium channels, which are required for their targeting to the axon initial segment.^164^

Notably, the palmitoylation process influences the localization of ZDHHC6, and the depalmitoylation of Cys328 renders it more susceptible to degradation by the ERAD pathway.^165^ ZDHHC16-mediated SETD2 palmitoylation has been shown to promote DNA damage in EGFR-mutated glioblastoma.^166^ Additionally, ZDHHC20 palmitoylates EGFR on the C-terminal domain, leading to the activation of EGFR signaling, and knocking down *ZDHHC20* has been demonstrated to reduce the growth of existing tumors derived from KRAS-mutant lung cancer cells and increase the sensitivity of these cells to EGFR inhibitors.^167^ Conversely, ZDHHC20 also palmitoylates CD80 protein, protecting it from degradation and regulating its stability, which is crucial for T cell activation.^168^

In leukemic blasts, ZDHHC21 exhibits catalytic activity in the palmitoylation of AK2, resulting in the activation of OXPHOS. However, targeting ZDHHC21 has been shown to markedly eradicate AML blasts and enhance the efficacy of chemotherapy.^169^ Furthermore, ZDHHC21, functioning as a tumor suppressor, interacts with FASN and mediates its palmitoylation, leading to decreased FASN protein stability and inhibition of FA synthesis, thereby suppressing DLBCL cell growth.^170^ ZDHHC21 also mediates PLCβ1 palmitoylation in endothelial inflammation, where inflammation enhances PLCβ1 palmitoylation and signaling activity. Inhibition of ZDHHC21 attenuates the inflammatory response.^171^

### Enzymes for depalmitoylation: PPT1, PPT2, APT1, APT2, ABHD17

Given that palmitoylation is a dynamic and reversible modification process, cysteine deacylases are responsible for depalmitoylation.^172^ Unlike the large number of palmitoyltransferases, only a few depalmitoylases have been discovered, including palmitoyl protein thioesterase-1 (PPT1), PPT2, APT1, and APT2, as well as the recently named α/β hydrolase domain 17 (ABHD17) family of depalmitoylases.^173^ These enzymes may function at different stages of protein trafficking to promote PM delivery and residency (Table S2).

PPT1, the first identified depalmitoylation enzyme, is primarily located in lysosomes but also targets the Golgi apparatus, membrane rafts, and the nucleus.^174^ Its main function is to maintain the stability of membrane proteins, regulate the catalytic activity, and facilitate vesicle-mediated transport.^175^ For instance, mitochondrial ATP synthase F1 complex is one of the substrates of PPT1, and its depalmitoylation is indispensable for F1 proper localization.^176^ In addition, PPT1 affects the mitochondrial respiratory chain by regulating the function of complexes I and IV of the mitochondrial electron transport chain.^177^ PPT1 also participates in the regulation of V-ATPase function by affecting its trafficking to the lysosomal membrane, thereby regulating autophagy levels.^178^ Overexpression of PPT1 in neuroblastoma cells inhibited caspase-3 palmitoylation and activation, promoting the anti-apoptotic activity of AKT.^179^ Although PPT2 is a homolog of PPT1, the cellular phenotypes caused by *PPT1* mutation cannot be rescued by PPT2, likely due to its smaller lipid-binding pocket.

APT1 is primarily localized in mitochondria and plays a partial role in regulating mitochondrial S-palmitoylation. As a major depalmitoylating enzyme with strong depalmitoylation activity distributed throughout the cell,^180^ APT1 can catalyze its own depalmitoylation and that of APT2, mediating membrane-cytosol shuttling and facilitating steady-state membrane function and localization.^181^ ZDHHC3/7 mediates APT2 palmitoylation, allowing soluble APT2 to stably bind to the membrane and interact with potential substrates.^182^ APT2 shares high similarity with APT1, but their substrate specificity differs. For example, the depalmitoylation of BK channels is controlled by APT1 rather than APT2, whereas ZDHHC6 can be stabilized and depalmitoylated by APT2.^165^^,^^183^ Inhibition of APT1 is considered to enhance synaptic function, but its exact role in tumors requires further investigation.

ABHD17 enzymes undergo self-palmitoylation, typically at the N-terminal cysteine-rich domain, which is essential for their PM localization and interaction with substrates. For instance, the synaptic protein PSD-95 is rapidly depalmitoylated by ABHD17 after depolarization.^184^ However, the kinetics, substrate specificity, and other functional properties of ABHD17 enzymes, as well as the redundancy between isotypes and the physiological role of each enzyme, remain to be explored. Furthermore, we summarized the genetic mouse models for *ZDHHC*s and depalmitoylates as below (Table 2).

**Table 2:** Genetic mouse models for *ZDHHCs* and depalmitoylates

Tissue specificity	Genes	Manifestations	References
Whole body	*Zdhhc2*^−/−^	decreased trend in Foxp3 protein expression in Tregs, increased the proportions of activated CD^4+^ and CD^8+^ T cells	Zhou et al.^185^
Whole body	*Zdhhc3*^−/−^ Zdhhc7^−/−^	decreased Foxp3 protein expression	Zhou et al.^185^
Whole body	*Zdhhc4*^−/−^	decrease FA uptake and develop severe hypothermia upon acute cold exposure	Wang et al.^52^
Adipose-specific	*Zdhhc5*^−/−^	decreased FA uptake activity	Wang et al.^52^
Whole body	*Zdhhc5*^−/−^	defected NLRP3 inflammasome activation *in vivo* decreased FA uptake activity in adipose tissues	Zheng et al.^186^
Whole body	*Zdhhc5*^−/−^	reduced NOD1/2 membrane association	Lu et al.^132^
Whole body	*Zdhhc6*^−/−^	elevated tumor diameter in DEN-induced HCC model	Zhou et al.^137^
Whole body	*Zdhhc7*^−/−^	impaired palmitoylation of Glut4 and decreased PM-associated Glut4 levels	Du et al.^187^
Whole body	*Zdhhc8*^−/−^	enhanced radio-sensitivity and inhibited tumor growth in a mesothelioma	Sudo et al.^139^
Whole body	*Zdhhc11*^−/−^	exhibited lower serum cytokine levels and higher lethality after HSV-1 infection	Liu et al.^144^
Whole body	*Zdhhc12*^−/−^	enhanced inflammatory symptoms and lethality following alum-induced peritonitis and LPS-induced endotoxic shock	Wang et al.^147^
Whole body	*Zdhhc13*^−/−^	generalized hypotrichosis with increased susceptibility to chemically induced skin carcinogenesis	Perez et al.^188^
Whole body	*Zdhhc13*^−/−^	generalized hypotrichosis, motor deficits, deficits in hindlimb muscle strength, robust gait anomalies	Napoli et al.^189^
Whole body	*Zdhhc15*^−/−^	increased novelty-induced locomotion, striatal dopamine content was reduced, and dopamine increased	Mejias et al.^190^
Whole body	*Zdhhc17*^−/−^	displayed striatal dysfunction, motor deficits, increased escape response	Sanders et al.^191^
Whole body	*Zdhhc17*^−/−^	reduced AKT phosphorylation along with reduced Akt palmitoylation, and protect HFMCD-induced NASH	Bu et al.^154^
Liver	*Zdhhc17*^−/−^	impaired AMPK activation and hepatic autophagy and caused a type 2 diabetes-like syndrome	Sun and Du^152^
Whole body	*Zdhhc18*^−/−^	exhibited prolonged survival and were less vulnerable to DNA viruses	Shi et al.^192^
Whole body	*Zdhhc20*^−/−^	reduced HCC formation, lesser liver nodules, lower biochemical markers of liver injury and inflammatory markers	Mo et al.^193^
Whole body	*Zdhhc21*^−/−^	marked resistance to injury, plasma leakage, and leukocyte adhesion decreased, ameliorated lung pathology, improved survival	Beard et al.^171^
Whole body	*Zdhhc21*^−/−^	decreased activation of the proximal TCR signaling components and suppressed cytoplasmic Ca^2+^ release in response to TCR stimulation	Bieerkehazhi et al.^194^
Murine forebrain	*Zdhhc21*^−/−^	displayed depression-like behavior	Gorinski et al.^195^
Whole body	*Zdhhc24*^−/−^	reduced AKT phosphorylation along with reduced AKT palmitoylation, and protect HFMCD-induced NASH	Bu et al.^154^
Whole body	*Ppt1*^−/−^	downregulated in AMPK/SIRT1/PGC-1α signaling pathway, increased p-S6K1 levels	Wei et al.^196^
Whole body	*Ppt1*^−/−^	more sensitive to mitochondrial complex I inhibition	Meyer et al.^197^
Whole body	*Ppt2*^−/−^	manifests as a neurodegenerative disorder with visceral features	Gupta et al.^198^
Endothelial cell	*Apt1*^−/−^	vessel area was reduced and intima fibronectin increased, decreased hindlimb perfusion recovery after femoral artery ligation	Wei et al.^199^

## Palmitoylated proteins and their major roles in cancer

Protein palmitoylation is a highly conserved PTM present in all eukaryotes, playing a pivotal role in regulating protein stability, subcellular localization, membrane trafficking, interaction with effector proteins, enzymatic activity, and a variety of other cellular processes. Unlike other static lipid modifications, the unique reversibility of palmitoylation provides an important mechanism for rapidly mediating protein transport between organelles and regulating protein dissociation or aggregation in membrane compartments. Approximately 10% of human proteins are palmitoylated in the SwissPalm database, collectively constituting the “palmitoylome.”^200^ So far, more than 600 substrates have been identified and experimentally determined, and the majority of them including ZDHHCs and palmitoylated residues and their functions in cancer have been summarized (Table S3).

### The functions of palmitoylation in protein regulation

#### Palmitoylation regulates protein subcellular localization

The fundamental role of palmitoylation is to alter the hydrophobic properties of substrate and influence their membrane localization (Figure 2). The trafficking of H-Ras and N-Ras requires palmitoylation, with palmitoylated cysteine residue sufficient for the translocation of N-Ras from the ER to the PM.^201^ H-Ras can be palmitoylated on either or both of two cysteine residues, which are not equivalent in terms of trafficking.^202^ Palmitoylation further enhances membrane protein binding, as seen with MCIR and CD36.^52^^,^^125^ It also blocks E3-mediated degradation or endocytosis, as observed with PD-L1 and EGFR,^142^^,^^203^ or enhances activation by membrane proteins, such as STAT3.^159^ Thus, reduced palmitoylation may allow protein dissociate from the PM into the cytoplasm or nucleus (Figure 2A). Lck is another dually palmitoylated protein involved in TCR signaling and T cell development, and the stable association with PM is mediated by palmitoylation at two cysteines.^204^ CD36 undergoes ZDHHC4- and ZDHHC5-mediated palmitoylation on four cytosolic cysteines, allowing proper targeting to the PM and maintaining its cell-surface location by protecting it from depalmitoylation, which is required for CD36-dependent FA uptake.^52^ Recently, we reported that ZDHHC17/24-mediated palmitoylation of AKT and promotes AKT membrane localization in a PI3K-PIP3-independent manner, resulting in AKT activation and HCC.^154^

NLRP3 is localized on the membrane of the *trans*-Golgi network (TGN) during the priming stage, followed by transient association with mitochondria during early activation. NLRP3 is then recruited to dispersed TGN or endosome, and eventually to mitochondria for inflammasome assembly. Recent studies reported that the palmitoylation mediated by ZDHHC1/7 is crucial for precise localizing at each step and ultimately full NLRP3 activation.^205^^,^^206^ Administration of 2-BP hinders the co-localization of NLRP3 in dTGN, endosomes, and the mitochondria marker TOMM20. Conversely, increased co-localization was observed with PA and Palm B treatments. Interestingly, palmitoylation at Cys958 could occur during the priming stage, facilitating NLRP3 localization to the TGN and transient targeting to mitochondria to enhance the formation of the double-ring cage. However, palmitoylation at Cys130 may be responsible for NLRP3 localization to dTGN and endosome membranes during the activation stage.^205^ Although mild inhibition of NLRP3-TGN co-localization was observed upon silencing *ZDHHC7*, the functional differences between ZDHHC1 and ZDHHC7 in regulating NLRP3 remain under investigation.

Under resting state, MAVS C-terminal tail-anchored mitochondrial outer membrane protein forms functionally essential prion-like aggregates upon activation by viral RNA-sensing RIG-I-like receptors, which required its palmitoylation by ZDHHC7 at Cys508. This palmitoylation stabilizes MAVS aggregation on the mitochondrial outer membrane, promoting subsequent antiviral signaling propagation.^207^

Furthermore, palmitoylation of proinflammatory cytokines such as TNF-α is required for precise localization in lipid rafts and efficient cleavage of their intracellular fragments.^208^ TNFR1 is also palmitoylated at multiple cysteine residues, facilitating its translocation to PM. Upon TNF-α binding, activated TNFR1 undergoes APT2-mediated depalmitoylation, resulting in translocation to another lipid raft, complex formation, and NF-κB activation.^209^ Palmitoylation of hexokinase 1 (HK1) in hepatic stellate cells facilitates HK1 secretion via large extracellular vesicles. In addition, TGF-β facilitates HK1 secretion by inhibiting ABHD17B-dependent depalmitoylation of HK1.^210^

#### Palmitoylation regulates protein interaction and conformation

Protein palmitoylation can enhance the binding affinity of modified protein to their binding partner on the PM (Figure 2B). Histone H3 undergoes palmitoylation, influencing the interaction between the nuclear membrane and chromatin or modify the stability of the H3/H4 tetramer.^211^ ZDHHC19 mediated palmitoylation of Smad3 promotes its interaction with EP300 and competitively reduces E2F4 inhibition, thereby activating the TGF-β/Smad signaling pathway.^212^ STING palmitoylation is important for TBK1 recruitment and activation, triggering the innate immune response.^213^ ZDHHC5-mediated NLRP3 palmitoylation at the LRR domain promotes NLRP3-NEK7 interaction, facilitating subsequent inflammasome assembly and activation. However, the abortion of ZDHHC5 blocks NLRP3 oligomerization and NLRP3-NEK7 interaction, abrogation of caspase-1 activation, IL-1β/18 release, and gasdermin D (GSDMD) cleavage.^186^ Another study showed that palmitoylation inhibits DNA binding to cGAS, which might be due to the ZDHHC18-mediated palmitoylation hindering cGAS dimers formation.^192^ Similarly, the NOD1/2 mutant proteins fail to form phagosomes and are depleted from the membrane fraction.

Palmitoylation can also modify the accessibility of vital active sites, altering protein conformation (Figure 2D). Caspase-6 (CASP6), a member of the cysteine protease family, is palmitoylated by ZDHHC17 at Cys264 and Cys277 near the start of the β sheet. Palmitoylation of CASP6 inhibits its activation via steric blockage of the substrate-binding groove and inhibition of CASP6 dimerization, both essential for CASP6 function.^214^ Palmitoylation of TEAD did not alter its localization but was required for TEAD binding to its transcriptional cofactor YAP and TAZ. Crystal structures reveal a deep, hydrophobic pocket in TEAD that accommodates palmitate binding, which allosterically regulates TEAD-YAP interaction and TEAD transcriptional activity.^215^

#### Palmitoylation regulates protein stability and degradation

Palmitoylation exhibits a protective effect against protein degradation by preventing ubiquitination, usually near the palmitoylation site, and sometimes by prompting protein membrane localization (Figure 2C). Recent studies of SNARE palmitoylation in *S. cerevisiae* and anthrax toxin receptor trafficking in mammalian cells have indicated that palmitoylation protects proteins from degradation by preventing their ubiquitylation.^216^^,^^217^ β2-Adrenergic receptor (β2AR) undergoes a dynamic cycle of palmitoylation, trafficking to the Golgi complex and returning to the PM, where palmitoylation may stabilize the receptor.^163^ The estrogen receptor ERα undergoes palmitoylation, protecting it from E2-dependent degradation and controlling ERα transcriptional activity by promoting its association with promoter and the nuclear matrix.^218^

Recently, palmitoylation has been demonstrated to function as a “brake” that shuts down overt NLRP3 inflammasome activation (Figure 2E). To this end, ZDHHC12-mediated NLRP3 palmitoylation prevents persistent inflammation by promoting NLRP3 degradation through the CMA pathway.^147^ Palmitoylation prevents CCR5 degradation, stabilizing CCR5, and the mutation of its residues leads to greatly reduced surface expression of CCR5.^219^ Additionally, palmitoylation of both PD-1 and PD-L1 prevents their degradation and promote tumorigenesis.^142^^,^^220^

### Palmitoylation-mediated regulation of the oncogenic and tumor-suppressive proteins

#### Palmitoylation in the MAPK pathway

**Figure 3:**
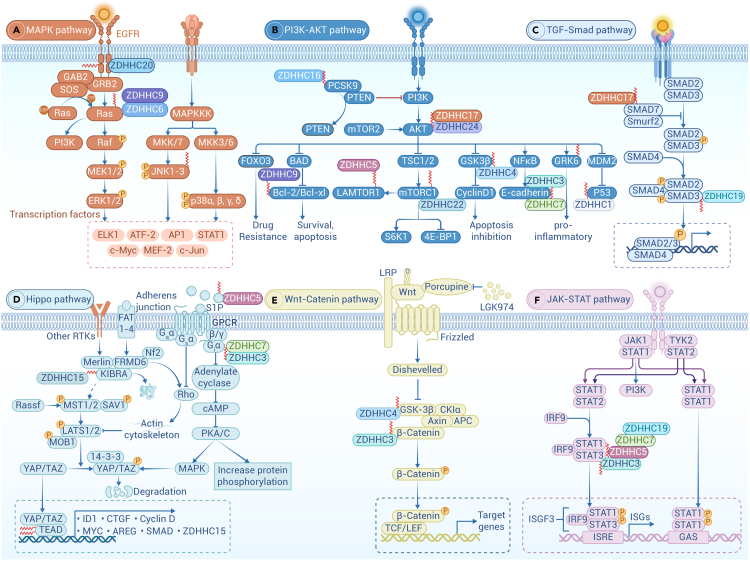
Major pathways regulated by palmitoylated proteins Illustration of palmitoylation-mediated regulation in some essential signal pathway, including MAPK pathway (A), PI3K-AKT pathway (B), TGF-β-SMAD pathway (C), Hippo and GPCR signaling pathway (D), Wnt signaling pathway (E), and JAK-STAT pathway (F).

One of the most relevant factors in tumorigenesis is the aberrant activation of the oncogene Ras, which mediates extracellular signaling and promotes tumor growth. Palmitoylation is required for specific isoforms of Ras to localize to the PM, where it becomes activated and signals downstream effectors in the MAPK signaling pathway, regulating cell proliferation.^217^ In the cytosol, the farnesyl transferase enzyme modifies Ras proteins at a conserved C-terminal CAAX sequence. However, farnesylated Ras exhibits poor membrane affinity, therefore, proper delivery of these Ras proteins to the PM through the secretion system critically depends on their palmitoylation at the Golgi.^201^ Overexpressing ZDHHC9 promotes Ras PM localization and transformation of mammalian cells.^221^ Palmitoylated Ras proteins move between compartments on vesicles, whereas depalmitoylated Ras proteins move rapidly through the cytoplasm in a vesicle-independent manner.^222^ GCP16 stabilizes ZDHHC9 for N-Ras and H-Ras palmitoylation,^223^ and RAB27B mediates N-Ras palmitoylation and PM localization by recruiting ZDHHC9. The ZDHHC9-GOLGA7 complex is essential for N-Ras trafficking, leading to its accumulation in the Golgi (Figure 3A).^224^

Newly synthesized N-Ras is initially farnesylated in the cytoplasm and then palmitoylated by palmitoyl acyltransferases at the ER and Golgi surfaces. Artemisinin (ART), a clinically approved antimalarial endoperoxide, covalently binds and inhibits ZDHHC6, reducing palmitoylation of N-Ras, disrupting its subcellular localization, and attenuating the downstream pro-proliferative signaling cascades.^225^ ABHD17 is required for N-Ras depalmitoylation and re-localization to internal cellular membranes.^173^

Breast cancer cells expressing palmitoylation-deficient EGFR^C1025A^ showed increased cell death after gefitinib treatment compared with WT, suggesting that the sensitization of triple-negative breast cancer cells to gefitinib is mediated by non-palmitoylated EGFR.^226^

#### Palmitoylation in PI3K-AKT pathway

The PI3K-AKT pathway is a crucial cellular signaling axis that regulates cell survival, growth, and metabolism. We have reported the indispensable role of AKT palmitoylation in the development of MASLD and HCC.^154^ A recent study showed that LPS-induced increased palmitoylation of GRK6 promotes its translocation to cell membranes, enhancing inflammatory responses via the PI3K/AKT signaling pathway (Figure 3B).^227^

Palmitoylated PCSK9 increased the binding affinity between PCSK9 and PTEN, inducing lysosome-mediated PTEN degradation and subsequent AKT activation.^228^ As the first substrate of AKT, GSK3β is involved in tumor formation and progression and acts as a tumor suppressor because its activation accelerates the degradation of oncogenes such as cyclin D1 and c-Myc.^229^ One recent study found that GSK3β palmitoylation, mediated by ZDHHC4, decreases *p*-Ser9 and increases *p*-Tyr216, leading to continuous activation of GSK3β, promoting tumorigenicity of glioblastoma stem cells.^131^

mTORC1 signaling is dynamically regulated by palmitoylation, which targets key proteins such as LAMTOR1 and mTOR to membranes. LAMTOR1, a component of the regulatory complex required for the lysosomal membrane localization of mTORC1, is directly palmitoylated, and its palmitoylation rapidly increases upon amino acid stimulation. Conversely, mTOR palmitoylation decreases upon activation of mTORC1.^230^ This dynamic palmitoylation suggests that it is not merely permissive for mTOR activation but actively involved in mTORC1-dependent signaling, which is essential for mTORC1 activation in response to nutrients and growth factors.

#### Palmitoylation in the TGF-β-SMAD pathway

Palmitoylation plays a potential role in the TGF-β/SMAD pathway, affecting the localization, activity, and function of key proteins within this signaling cascade, such as the aforementioned Smad3 palmitoylation by ZDHHC19 in the heterogeneity of glioblastoma stem cell subsets. Another inhibitory Smad, Smad7, is palmitoylated by ZDHHC17, promoting its translocation from the nucleus to the cytoplasm, enhancing protein stability, and increasing its inhibitory effect on TGF-β/SMAD signaling (Figure 3C).^231^

#### Palmitoylation in the Hippo pathway

TEAD palmitoylation plays a key role in the Hippo signaling pathway. TEAD binds to the coactivators YAP and TAZ, regulating the transcriptional output of the Hippo pathway and impacting organ size control and tumorigenesis. TEAD undergoes auto-palmitoylation at conserved cysteine residues under physiological conditions, a process essential for its binding to YAP and TAZ.^215^ This modification is required for proper TEAD folding and protein stability, as well as the full transcriptional regulation of the Hippo pathway. However, TEAD palmitoylation is actively regulated by cell density independent of Lats, the key kinase of the Hippo pathway.^232^ During the process, FASN and ACC in *de novo* palmitate biosynthesis are reduced by cell density in an Nf2-Merlin-dependent manner. Dysfunction in TEAD palmitoylation leads to instability and proteasomal degradation via the E3 ubiquitin ligase CHIP (Figure 3D).^232^

Scribble (SCRIB) regulates cell polarity, and has also been identified as a tumor suppressor. A recent study identified two membrane terminal cysteines in SCRIB that are palmitoylated by ZDHHC7, regulating the ability of the protein to localize to cell junctions.^124^ Correct localization of SCRIB at the PM activates the Hippo kinase cascade and inhibits MAPK and AKT signaling.^124^ Abortion of palmitoylation results in mislocalization of SCRIB, blocking its ability to suppress oncogenic signaling pathways (Hippo, PI3K/AKT, and MAPK) and activating HRasV12-mediated cell invasion.

#### Palmitoylation in the GPCR signaling

GPCRs represent the largest family of integral membrane proteins, detecting a wide variety of ligands, including peptides, hormones, lipids, and nucleotides. The G protein α subunit can be palmitoylated by ZDHHC3/7, which is required for PM localization and PM-Golgi shuttling.^233^ ZDHHC5 can promote immune response via S1PR palmitoylation, which is required for efficient S1PR signaling by affecting receptor internalization and association with the Gβγ subunits,^234^ although S1PR palmitoylation is not involved in cell surface expression of S1PR.^235^ RGS protein regulates trimeric G protein signaling and is involved in inflammation and immunity. Many RGSs undergo palmitoylation, such as RGS10, which is important for its activity in inhibiting Gsα-mediated signaling,^236^ and palmitoylation of RGS16 significantly affects GTPase accelerating (GAP) activity and Gi-coupled signaling regulation (Figure 3D).^237^

#### Palmitoylation in the Wnt signaling pathway

Wnt signaling is a conserved signaling pathway in which extracellular Wnt proteins bind to receptors on the cell membrane through diffusion, activating downstream intracellular signal transduction and intranuclear transcriptional regulation. Unlike conventional palmitoyl transferase, the *O-*acyltransferase Porcupine is responsible for palmitoleoylation by transferring the unsaturated FAs produced by SCD to Wnt, conferring functional activity on the Wnt protein family and triggering signaling.^238^ It has been shown that Wnt3a is acylated on two conserved residues: Cys77 is acylated by PA, affecting its activity, and Ser209 is linked to palmitoleic acid, affecting its secretion.^239^ Treatment of cells with SCD inhibitors blocked the incorporation of palmitate analogs into Wnt3a and Wnt5a, reducing Wnt secretion and Wnt signaling transduction.^240^ Interestingly, WNT1 is sufficient for PORCN-dependent palmitoylation of Ser224, but not cysteine.^241^ PORCN specifically acylates Wnt ligands, leading to a strategy for drugs that specifically inhibit the enzyme as a possible treatment against cancerous processes. Indeed, the Porcupine inhibitor LGK974 occupied the palmitoleoyl-CoA binding site to downregulate the Wnt signaling pathway, providing evidence for anti-cancer treatment (Figure 3E).^238^

### Functions of palmitoylated proteins in cell death control

**Figure 4:**
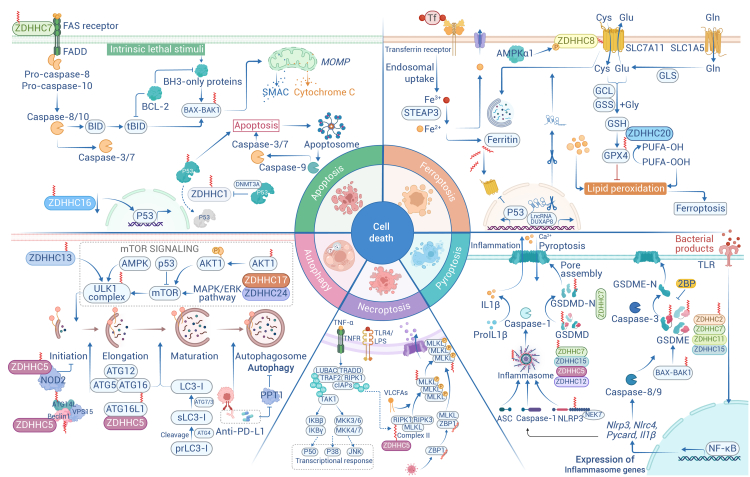
Protein palmitoylation in tumor cell deaths Illustration of palmitoylation in regulating multiple levels of cell death, including autophagy, apoptosis, necroptosis, pyroptosis, and ferroptosis. On the one hand, palmitoylation may arrest the cell cycle by affecting p53 and, on the other hand, it can also bind to death receptors on the cell surface to trigger key programmed cell death pathways and become a pro-apoptotic factor. Palmitoylation of Beclin 1 promotes the formation of an autophagic complex containing ATG14L, which enhances lipid kinase activity in response to lysosomal autophagy initiation. In addition, ULK1 and p62 palmitoylation can also occur through autophagy. Palmitoylation of the important ferroptosis proteins SLC7A11 and GPX4 promotes cell death driven by lipid peroxidation. Cellular pyroptosis is caused by caspase activation and inflammasome formation, NLRP3 undergoes multiple modifications to achieve its full activity, and palmitoylation defects trigger massive pyroptosis. In addition, palmitoylation modification is essential for GSDM-DN to disrupt effective plasma membrane permeability and cytokine release.

In the field of oncology, aberrant cell death pathways can initiate and promote tumorigenesis and influence therapeutic outcomes. Palmitoylation plays a crucial role in regulating multifaceted cell death, including autophagy, apoptosis, necroptosis, pyroptosis, and ferroptosis (Figure 4).

#### Palmitoylation in autophagy

Autophagy is a conserved mechanism in eukaryotes characterized by the formation of an autophagosome, the process is regulated by the AKT-mTOR pathway and autophagy-related genes.^242^ The AKT-C344 mutation blocks the palmitoylation and affects the phosphorylation of its key sites T308 and T450, increasing its recruitment tendency to co-localize with lysosomes in response to autophagy.^243^ Beclin 1, a central player in autophagy initiation, has been implicated in a variety of diseases through its regulation of autophagy and other cellular processes. Recently, the reduction of ZDHHC5-mediated Beclin 1 palmitoylation has been implicated in the decline of autophagy observed with aging.^244^ Palmitoylation of Beclin 1 promotes the formation of the ATG14L-containing class III phosphatidylinositol 3-kinase complex I. This complex is crucial for autophagy initiation, as it enhances lipid kinase activity and strengthens the hydrophobic interactions between Beclin 1 and adapter proteins ATG14L and VPS15. *ZDHHC5* deficiency leads to a decline in autophagy with age, which contributes to the accumulation of toxic protein aggregates and exacerbates neurodegeneration in an autophagy-dependent manner.^244^ In addition, ATG16L1 palmitoylation, catalyzed by ZDHHC7, enhances the formation of ATG16L1-WIPI2B and ATG16L1-RAB33B complex on phagophore, thereby facilitating the LC3 lipidation process and autophagosome formation.^245^

Another latest study revealed that unc51-like autophagy activating kinase 1 (ULK1) is palmitoylated by ZDHHC13, which is required for autophagy initiation. The palmitoylated ULK1 was translocated to the autophagosome formation site upon autophagy induction, enhancing the phosphorylation of ATG14L to form autophagosome membrane lipids.^246^ Under conditions of energy deprivation or stress, AMPK is activated and phosphorylates ULK1 to initiate autophagy or directly phosphorylates p62, promoting its translocation to mitochondria and inducing mitophagy.^247^ Additionally, AMPKα palmitoylation, mediated by ZDHHC17, enhanced AMPK activation, and inactivation of ZDHHC17 in the mouse liver impaired autophagy and decreased ULK1 phosphorylation.^152^

Palmitoylation of p62, catalyzed by ZDHHC19 and APT1, enhances its binding to autophagosome membranes and promotes the incorporation of p62 bodies into autophagosomes. This process increases the affinity of p62 for microtubule-associated protein LC3-positive membranes, facilitating the localization of p62 droplets to autophagic membranes and ultimately leading to the efficient degradation of p62 and its cargo proteins via the autophagy pathway, highlighting the importance of p62 palmitoylation in selective autophagy.^248^ While PPT1 is the primary de-palmitoylating enzyme on lysosomes, overexpression of PPT1 confers resistance to autophagy and apoptosis. Studies have found that the combined use of anti-PD-1 antibodies and PPT1 inhibitor HCQ or DQ661 can induce autophagy *in vivo* in melanoma.^249^^,^^250^

#### Palmitoylation in apoptosis

Apoptosis is crucial for maintaining cellular equilibrium in multicellular organisms by selectively eliminating damaged or surplus cells. Internal stressors such as DNA damage or oxidative stress initiated the p53 protein to promote apoptosis, preventing the proliferation of severely damaged cells.^251^ It has been demonstrated that reduced expression of ZDHHC16 leads to p53 activation and cell-cycle arrest in EGFR-altered GBM.^166^ Further research has revealed that palmitoylation inhibition activates the ER stress response and downstream signaling targets, leading to apoptosis. For example, a mutation in XBP1 decreases its palmitoylation and enhances the accumulation of SUMOylated XBP1, which in turn suppresses its transcriptional activity.^252^ The BCL-2-associated X (BAX) protein shuttles between the cytosol and mitochondria, promoting the release of cytochrome *c* and ultimately resulting in apoptosis. Its pro-apoptotic function is regulated by palmitoylation at Cys126, which is crucial for BAX mitochondrial translocation, oligomerization, and caspase activation. Reduced BAX palmitoylation decreases its mitochondrial targeting and apoptotic capacity. However, malignant tumor cells often show decreased BAX palmitoylation, consistent with their reduced proapoptotic activity.^253^

Additionally, extracellular death ligands trigger a key programmed cell death pathway by binding to death receptors on the cell’s surface. TNFR1 and FAS, death receptors belonging to the TNFR superfamily, initiate apoptosis by recruiting FAS-associated death domain protein and procaspase-8. A previous study confirmed that ZDHHC7 palmitoylates FAS (CD95) at Cys199, preventing its degradation via lysosomes and promoting its assembly into macroaggregates at the cell membrane, FAS internalization, and thereby enhancing apoptosis.^254^^,^^255^ Another pathway, mediated by CHOP/GADD153, depends on ER stress activation in response to the accumulation of misfolded proteins or calcium imbalance.^256^ Notably, a recent report showed that the ZDHHC5-GOLGA7 complex is required for small-molecule CIL56 to induce an unconventional form of nonapoptotic cell death.^257^ The anti-apoptotic kinase AKT also undergoes ZDHHC17/24-mediated palmitoylation, markedly blocking drug-induced cancer cell apoptosis.^154^

#### Palmitoylation in necroptosis

RIPK1 functions as a molecular switch; when caspase activity is inhibited, RIPK1 is activated to interact with RIPK3, leading to the formation of a RIPK1/RIPK3 complex necrosome, which activates mixed lineage kinase-like protein (MLKL), facilitating the execution of necroptosis. It has been reported that palmitoylation of MLKL and *p*-MLKL play an important role in necroptosis, particularly as very-long-chain FAs promote membrane localization and protein stability of MLKL and pMLKL.^258^^,^^259^ The molecular mechanism linking palmitoylation of RIPK1 to necroptosis has been elucidated through recent research, identifying palmitoylation as a crucial modification that licenses the kinase activity and cytotoxic potential of RIPK1 in the TNF pathway when the protective checkpoint is disabled.^260^ ZDHHC5 was identified as the primary enzyme responsible for RIPK1 palmitoylation upon TNF stimulation, dependent on K63-linked ubiquitination of RIPK1 within complex I. Mechanistically, RIPK1 palmitoylation promotes the kinase activity by promoting the homo-interaction of its kinase domain, in turn promoting cell death.^260^ Interestingly, both WT and phosphorylated MLKLs undergo ZDHHC5-mediated palmitoylation during necroptosis, thus perturbing its biological functions in cells that will likely rescue cell death from necroptosis.^258^

#### Palmitoylation in ferroptosis

Ferroptosis is a form of iron-dependent cell death driven by lipid peroxidation.^261^ Sorafenib, a versatile tyrosine kinase inhibitor, induces iron enrichment but faces the challenge of drug resistance. New findings reveal that the lncRNA DUXAP8 plays a role in sorafenib resistance by suppressing ferroptosis. Mechanistically, DUXAP8 promotes the palmitoylation of solute carrier family 7 member 11 (SLC7A11) and prevents its lysosomal degradation, thereby enhancing the function of SLC7A11 and inhibiting iron accumulation.^262^ Additionally, evidence has shown that SLC7A11 is palmitoylated by ZDHHC8 at C327, decreasing its ubiquitination level in GBM, while AMPKα1 phosphorylates ZDHHC8 to strengthen their interaction.^263^ It has been reported that ZDHHC7 may regulate the nuclear localization of YAP1 in ovarian clear cell carcinoma, and the inhibition of ZDHHC7 enhances ferroptosis by activating YAP1, although it is uncertain whether YAP1 is palmitoylated.^264^

#### Palmitoylation in pyroptosis

Pyroptosis is a form of programmed cell death initiated by the activation of inflammatory caspases, which cleave gasdermin (GSDM) family proteins, releasing an N-terminal fragment to form pores in the PM, resulting in cell death and release of proinflammatory cytokines.^265^ Recent studies have revealed that the palmitoylation at Cys191 in GSDM-D plays a pivotal role in the membrane translocation and oligomerization of GSDM-DN. This modification is crucial for the efficient permeabilization of the PM and cytokine release, which is a hallmark event during pyroptosis.^266^^,^^267^ Given this new insight into GSDM biology, control of GSDMD activation through palmitoylation-depalmitoylation during pyroptosis may offer an additional target for modulating immune activity in inflammatory diseases and tumors.^268^ Besides, studies have also identified that chemotherapy drugs can induce pyroptosis in cancer cells through the BAK/BAX-caspase-3-GSDME signaling pathway.^269^ Specifically, GSDME is palmitoylated on its C-terminal (GSDME-C), which facilitates the dissociation of GSDME-C from GSDME-N and leads to an increase in chemotherapy drug-induced pyroptosis. Of course, the palmitoylation inhibitor 2-BP has been shown to inhibit the pyroptosis induced by chemotherapy drugs.^270^

Another key component in the inflammasome is NLRP3, and the importance of palmitoylation for associated proteins has been shown in our recent work.^271^ Upon priming and activation, NLRP3 undergoes multiple PTMs to reach its full activity, ZDHHC12, as the *S*-acyltransferase for NLRP3 palmitoylation, promotes its autophagy degradation pathway. Conversely, defective palmitoylation results in overt NLRP3 inflammasome activation, triggering massive pyroptosis.^147^

## Functions of protein palmitoylation in regulating immune responses and TME

The regulation of palmitoylation is crucial for immune cells, as it is essential for the proper functioning of numerous surface receptors and signaling proteins, including adhesion, transmigration, chemotaxis, phagocytosis, pathogen recognition, T cell receptor (TCR), and BCR signaling activation, cytotoxicity, cytokine production, and exhaust.

### Palmitoylation in innate immune cells

**Figure 5:**
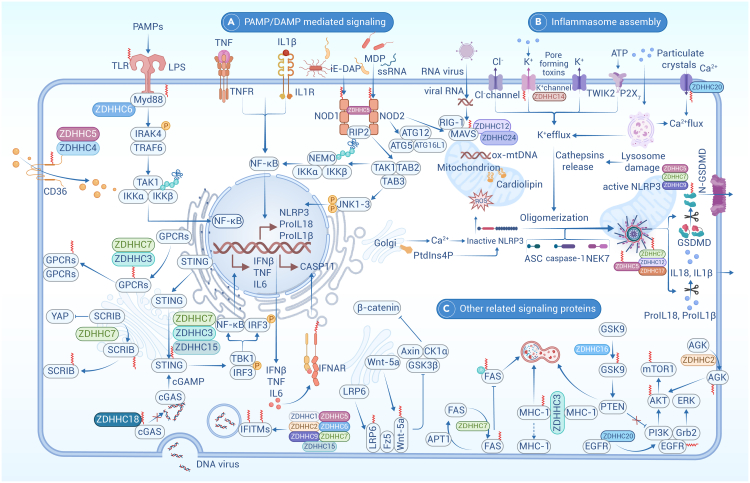
Palmitoylation in innate immunity Palmitoylation modulates multiple innate immune signaling pathways mediated by PAMPs, DAMPs, viruses, and bacteria. (A) Palmitoylation facilitates the interaction between innate immune receptors/membrane receptors and adaptor proteins. Palmitoylated MyD88 forms complexes with IL-1 receptor-associated kinases (IRAKs) to activate downstream signaling, enabling NF-κB nuclear translocation. Palmitoylation of NOD1/2 recruits them to bacteria-containing phagosomes, where TAK1 activation drives cytokine transcription and antiviral protein production. STING undergoes palmitoylation during its trafficking from the ER to the Golgi, enabling aggregation and recruitment of TBK1 and IRF3 for downstream signaling. Innate immune cells utilize palmitoylation to regulate membrane channel activity, promote inflammasome assembly, and trigger cytokine release for immune cell recruitment. (B) Palmitoylation enhances inflammasome assembly (ubiquitous in innate immunity) by modulating ion channel states and amplifying immune responses. (C) Palmitoylation alters protein stability or activity, blocking degradation or activating signaling cascades (e.g., EGFR, N-Ras/H-Ras, GPCRs, AGK-AKT-mTORC1, FAS, and SIRP pathways).

The innate immune system acts as the primary defense against pathogenic invaders and plays a crucial role in maintaining tissue homeostasis. Recent studies have shed light on the role of palmitoylation in modulating these innate immune responses (Figure 5).

#### Palmitoylation in neutrophils

Neutrophils, the most abundant type of white blood cells circulating in the body, are among the first responders at the site of infection and contribute to the initiation and spread of the inflammatory cascade. Following neutrophil activation, the recruitment cascade begins with selectin-mediated slow rolling, followed by β2-integrin-mediated firm adhesion and PECAM-1 mediated transmigration across the endothelium.^272^ Recent studies showed that P-selectin can undergo palmitoylation, and depalmitoylation by APT1 greatly inhibits the expression of P-selectin and thrombus formation, potentially affecting neutrophil recruitment.^273^ Additionally, the palmitoylation of PECAM-1 is required for its lipid raft localization.^274^ Another study showed PECAM-1 is palmitoylated by ZDHHC21, and knockdown of *ZDHHC21* leads to reduced PECAM-1 at the endothelial cell surface.^275^

Compared with other immune cells that primarily signal through membrane receptors, the migration of neutrophils to sites of infection or injury is particularly important. Rac1, a member of the small Rho GTPase family, is essential for neutrophil motility. Palmitoylation-defection Rac1 reduces its ability to load GTP and decreases lipid raft partitioning, exhibiting spreading and migration defects.^276^

#### Palmitoylation in NK cells

Natural killer (NK) cells are broadly distributed innate cytotoxic lymphoid cells. In addition to producing lytic granules containing an arsenal of molecules that can induce cell death, they also express several TNF superfamily members, such as FASL and TRAIL, inducing apoptosis of target cells via binding to their corresponding receptors.^277^ Moreover, NK cells can shape the immune response by interacting with dendritic cells (DCs), macrophages, and T cells.^278^ NKG2D mediates the cytotoxicity of NK cells as an activating receptor expressed on NK cells.^279^ It is unknown whether NKG2D itself can be palmitoylated, but its ligand MHC-class-I-related chain A (MICA) has been reported to undergo palmitoylation at Cys306 and Cys307.^280^ Soluble MICA is a negative regulator of NKG2D, and deficiency of palmitoylation greatly reduces the production of soluble MICA, further promoting the activation of NK cells.^280^

#### Palmitoylation in monocytes and macrophages

Monocytes circulate and fight pathogens through phagocytosis, differentiating into macrophages or DCs once recruited to the inflammation sites. They also produce inflammatory mediators and act as antigen-presenting cells (APCs).^281^ A study has reported that 48% of palmitoylated proteins in macrophages are localized in lipid rafts and participate in various monocyte/macrophage functions.^282^ For example, Plscr3 was palmitoylated for mitochondrial targeting and pro-apoptotic function,^282^ and palmitoylation of TNFR1 is required for its PM localization in human monocytic cells.^209^

Palmitoylation is involved in LPS-induced macrophage activation. For instance, Src family kinase Fyn, which inhibits TLR4, requires palmitoylation at Cys3 for its lipid raft localization and activity.^283^ LPS stimulation enhances Fyn palmitoylation in lipid rafts, where it suppresses cytokine production via NF-κB and IRF3 pathways.^283^ CCR5, a chemokine receptor that regulates macrophage chemotaxis, is greatly reduced in surface expression when palmitoylation defective, leading to decreased binding ability with macrophage inflammatory protein-1β.^219^^,^^284^ CD36 is the primary scavenger receptor expressed on macrophages, and facilitates the phagocytosis of pathogens.^285^ Besides, activation of macrophages through phagocytic Fcγ receptors (FcγR) has been shown to require selenoprotein K (Selk).^286^ Upon deficiency of cofactor Selk, macrophages exhibit reduced membrane expression of CD36, likely due to the deletion of Selk connecting DHHC6 to the ER membrane, thereby reducing the DHHC6-mediated palmitoylation of CD36 in macrophage ER.^287^ Alongside its role as an adhesion molecule, CD44 acts as an accessory phagocytic receptor, with its palmitoylation essential for lipid rafts and endocytosis of hyaluronan by macrophages in a CD44-dependent manner.^288^

#### Palmitoylation in DCs

DCs, as the most potent APCs, are specialized to recognize, phagocytize, process, and present antigens to both memory and T cells.^289^ Palmitoylation of TLR2 was identified in DCs, positively regulating TLR2 activity and promoting its trafficking to the cell surface. Blocking TLR2 palmitoylation inhibits microbial ligands-induced activation of NF-κB and production of IL-6 and TNF-α.^290^ TLR9 is S-palmitoylated by ZDHHC3 in the Golgi, regulating TLR9 trafficking to endosomes. Subsequent depalmitoylation by PPT1 facilitates the release of TLR9.^291^ The palmitoylation and depalmitoylation cycles directly modulate the secretion of IFNα by plasmacytoid DCs (pDCs) and TNF by macrophages, and inhibition of PPT1 attenuates systemic autoimmunity reaction.^291^

### Palmitoylation in innate immune response

Innate immune responses recognize pathogen-associated molecular patterns (PAMPs) and damage-associated molecular patterns (DAMPs) through pattern recognition receptors, activating immune cells to release inflammatory factors to regulate immune response and enhance immune cell function. Interferons, cytokines, and chemokines can recruit more immune cells to the site of infection to engulf and digest pathogens.^292^ Palmitoylation can enhance innate immune responses or suppress immune molecules by affecting the localization, stability, and protein-protein interaction of innate immune sensors and adapters.^293^^,^^294^

#### Palmitoylation in TLR and NOD signaling pathways

TLRs are transmembrane proteins that detect various PAMPs, such as viral nucleic acids and microbial polysaccharides. Previous studies have shown that TLR signaling can be activated by saturated FAs, even though FAs do not bind to TLR.^295^ Another study suggested that the adaptor protein MyD88 is palmitoylated by ZDHHC6, an essential signaling adaptor for most TLRs, which is required for the binding of IRAK4 to the MYD88 intermediate domain and downstream NF-κB signal activation.^138^ In fact, many isotypes of TLRs (TLR 2, 5, 6, 9, 10) have been shown to be palmitoylated.^290^ Although long-chain saturated FAs such as palmitate are not TLR4 agonists, they indirectly regulate palmitate-induced inflammation by altering gene expression and lipid metabolism.^295^

Lu et al. reported that ZDHHC5-mediated palmitoylation of NOD1/2 promotes NOD1/2 recruitment to proper membrane.^132^ On the other hand, ZDHHC5 can be recruited to large phagosomes, and its catalytic activity to modify more NOD1/2, upregulating their function to sense bacterial peptidoglycans and activation of NF-κB and MAPK signaling to mount an effective innate immune response.^132^ *NOD1/2* polymorphisms are involved in a variety of diseases, with the NOD2 short isoform variant NOD2s-R444C associated with heightened inflammation and an increased risk of inflammatory bowel disease. Mechanistically, the NOD2s-R444C mutation possesses an enhanced ability to combine with ZDHHC5, leading to impaired SQSTM1/p62-mediated autophagic degradation of NOD2 and excessive inflammation.^296^

#### Palmitoylation in the RLR-MAVS signaling pathway in sensing double-strand RNAs

Previously, we have found that ZDHHC24 catalyzes MAVS palmitoylation, boosting the TBK1-IRF3-interferon (IFN) pathway and leading to antiviral immune responses.^297^ Similarly, recent studies also showed that the palmitoylation of MAVS enables to stable its aggregation on the mitochondrial outer membrane, promoting subsequent propagation of antiviral signaling. Conversely, inhibition of MAVS palmitoylation increases the host susceptibility to RNA virus infection.^87^^,^^207^ Boosting MAVS palmitoylation by APT2 inhibitors could elevate IFN signaling and anti-double-strand RNA (anti-dsRNA) viral infections.^297^

#### Palmitoylation in the cGAS-STING signal pathway in sensing dsDNAs

The stimulator of IFN genes (STING) is central to the production of type I IFNs in response to infection with DNA viruses. ZDHHC18-mediated palmitoylation of cGAS at Cys474 reduces the interaction between cGAS and dsDNA, further inhibiting cGAS dimerization, thereby negatively regulating cGAS-mediated innate immunity.^192^ However, a recent study revealed that Cys404/405 are highly probable to be crucial for cGAS palmitoylation mediated by ZDHHC9, not ZDHHC18, and this modification promotes cGAS enzymatic activity.^298^ STING detects cyclic dinucleotides from bacteria and DNA viruses, triggering strong cytokine release. It has been reported that STING palmitoylation occurs in the Golgi compartment and is essential for STING activation,^213^ particularly for TBK1/IRFF3 recruitment and enhancement of the downstream IFN pathway to trigger the innate immune response.^299^ Blocking STING palmitoylation significantly reduces the production of type I IFN and IFN-induced CXCL10 and IL-6 in monocytes and macrophages following viral infection.^300^ Indeed, in an attempt to identify STING inhibitors by chemical screening, small-molecule inhibitors targeting the transmembrane cys91 were discovered, thereby blocking the activation-induced palmitoylation of STING.^301^

#### Palmitoylation in anti-viral response

IFN-α/β receptor (IFNAR) is crucial for antiviral defense, binding type I IFNs to fight virus infections.^302^ Binding to type I IFNs triggers IFNAR endocytosis and activates JAK kinases, leading to the transcription of IFN-stimulated genes. Palmitoylation is essential for the IFNAR-JAK-STAT pathway. The palmitoylation inhibitor reduces IFNAR1 endocytosis and STAT1 phosphorylation. Mutating the palmitoylation site prevents STAT1/2 phosphorylation and nuclear translocation but not IFNAR1 endocytosis or stability.^303^ Another study has showed that the binding of IFNGR1 with AP3D1 depends on palmitoylation, and sorting palmitoylated IFNGR1 to lysosomes for degradation, thereby blocking IFN-γ and MHC-I signaling integrity to enhance T cell immunity. Therefore, targeting IFNGR1 palmitoylation can enhance T cell immunity and sensitize checkpoint therapy in CRC.^304^

Palmitoylation of IFITMs, an INF-stimulated effector protein, induced by several ZDHHCs, has been well recognized for its antiviral activity.^305^ IFITM3 is located on endocytic vesicles that fuse with invading virus particles and enhances the transport of targeted lysosomes. Although palmitoylation-deficient IFITM3 does not affect its expression or subcellular localization in DC, its ability to aggregate with viral particles is greatly reduced and its antiviral activity is impaired.^305^^,^^306^

### Palmitoylation in adaptive immune response

Unlike the rapid innate immune response, the adaptive immune response mainly involves T and B cells, which recognize specific antigens to produce specific immune responses and generate long-term protection due to memory T and B cells.

#### Palmitoylation in T cells

T cells participate in and coordinate a variety of adaptive immune responses. Several palmitoyl proteomic studies reveal that, although the TCR itself might not undergo palmitoylation, TCR co-receptors CD4, CD8, Src family kinases Lck and Fyn, and adaptor proteins, linker for activation of T cells (LAT) and PAG, are all palmitoylated.^307^ Among the PATs, ZDHHC21 was identified as required for the initiation of the TCR signaling pathway without effecting the development of regulatory T cells (Tregs) in the thymus.^194^ Disruption of the binding domain of ZDHHC21 prevents the differentiation of peripheral CD4^+^ T cells into effector helper T cells, which is essential for T cell-mediated immunity. Similarly, 2-BP disrupts signaling protein localization and blocks TCR signaling.^308^

Although research on CD4 palmitoylation has focused on the role of lipid raft targeting, the definitive effects of CD4 palmitoylation on T cell signaling.^309^ In the case of CD8, palmitoylation is demonstrated to occur on CD8α/β heterodimer in humans, resulting in a reduction of Lck activation, Ca^2+^ induction, and recruitment of the TCR to detergent-resistant membranes in mice, while palmitoylation in human CDα/β does not appear to play a role in this.^310^ LAT is an essential scaffolding protein involved in T cell development and activation, and it is phosphorylated predominantly by ZAP-70 kinase upon TCR ligation. Recent studies demonstrated that LAT palmitoylation induces its sorting from the Golgi compartment to the PM.^311^ Fyn, another highly expressed Src family kinase in T cells, can be activated by Lck. Similar to Lck, the attached palmitate on Cys3 is important for lipid raft association and downstream signaling.^312^ The co-aggregation of TCR and CD4 leads to the activation of Lck outside the lipid rafts, followed by its translocation to the lipid rafts, where it activates the co-localized Fyn.^313^

PD-1 is expressed on the surface of activated T cells as an inhibitory receptor, while its natural ligands PD-L1 and PD-L2 are mainly expressed in APCs and tumor cells. Recent studies discovered the expression of PD-1 in melanoma and HCC, as well as its pro-tumorigenesis function.^314^^,^^315^ Similar to PD-L1, PD-1 also can be palmitoylated by ZDHHC9, which promotes the trafficking of PD-1 to the recycling endosome and prevents its lysosome-dependent degradation, thereby activating mTOR signaling and tumor cell proliferation.^220^

CD80, belonging to the B7 family transmembrane glycoprotein, has emerged as a crucial molecule in T cell modulation via cognate interactions with the CD28 or CTLA4 to generate a second signal. ZDHHC20 as a bona fide palmitoyltransferase, determines the palmitoylation level of CD80. Palmitoylation prevents CD80 protein from ubiquitination degradation, regulates its stability, and ensures its accurate PM localization.^168^

ORAI1 channels are responsible for Ca^2+^ fluxes at the immune synapse (IS) between T cells and APCs. A study showed that ZDHHC20 mediated palmitoylation of the ORAI1 at residue Cys143 promoted TCR recruitment to the synapse and signaling transduction at the IS.^316^ Upon TCR activation, intracellular signaling effector IP3 triggers Ca^2+^ release from ER stores via binding to IP3Rs on the ER membrane. IP3Rs are palmitoylated by ZDHHC6/Selk complex in ER.^317^

#### Palmitoylation in B cells

B cells are specialized in producing highly specific antibodies and cytokines in response to various antigens in adaptive immunity. BCR signaling is amplified and prolonged, requiring co-ligation to the CD19/CD21/CD81 co-receptor complex. CD81 is a well-established palmitoylation substrate at multiple membrane cysteines.^318^ The palmitoylation of CD81 promotes the lipid raft association of BCR and co-receptor ligation, altering interaction with other proteins and affecting the ability of CD81 to recruit the downstream signaling molecule 14-3-3.^319^ Another raft localization protein, HGAL, facilitates interaction with Syk and modulation of the BCR activation and signaling. However, the mutation at palmitoylation sites cannot facilitate the translocation of HGAL into lipid rafts and fails to promote the phosphorylation of Syk and downstream Ca^2+^ influx.^320^

Fcγ receptors constitutively express on the surface of multiple immune cells, such as neutrophils upon activation, enabling them to recognize antigens via immunoglobulin-mediated interactions and regulate neutrophil functions. Therefore, Fcγ receptors serve as a crucial connection between innate immune effector cells and adaptive immune responses. It has been shown that the FcγRII (CD32) undergoes palmitoylation at Cys208, regulating the lipid raft targeting ability and affecting downstream signaling.^321^

### Palmitoylation in TME

**Figure 6:**
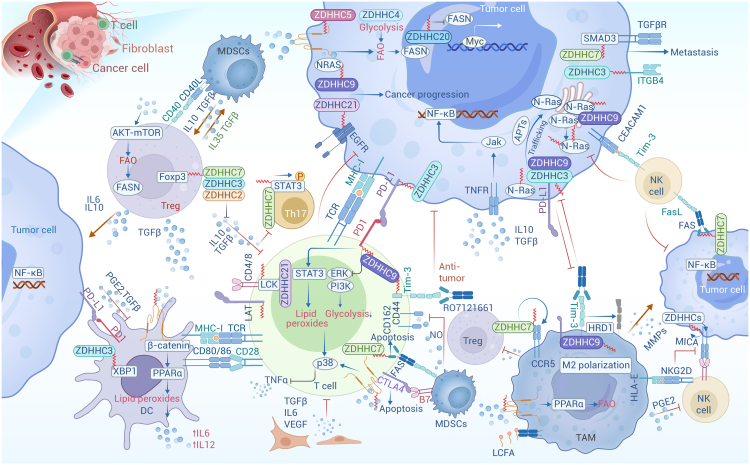
Palmitoylation in communications of TMEs Illustration of the functional roles of protein palmitoylation in the tumor microenvironment. Palmitoylation can regulate the expression and activity of immune inhibitory molecules, such as PD-L1, TIM-3. Palmitoylation can enhance the stability and activity of PD-L1 on the cell membrane, thereby strengthening its ability to suppress immune responses. Protein palmitoylation in T cell activation and immune regulation. Palmitoylation is essential for the proper functioning of the T cell receptor (TCR) signaling pathway. CD4 and CD8 palmitoylation is required for effective TCR signaling. Palmitoylation regulates the expression of Foxp3 target genes by affecting its DNA binding ability. Increased expression of these molecules could further enhance the immunosuppressive capacity of Tregs. Palmitoylation can also promote the secretion of immunosuppressive molecules, such as IL-10 and TGF-β. In addition, palmitoylation enhances immune escape to tumor cells by affecting immunosuppressive cells.

The TME is complex and heterogeneous. Protein palmitoylation contributes to the formation of an immunosuppressive TME by regulating the activation, infiltration, and antigen presentation of immune cells. The microenvironment can provide survival signals that protect cancer cells from dying, or it can be altered by the cancer cells to create a niche that supports their growth and spread. This complex interplay can lead to the development of more aggressive tumor phenotypes and contribute to tumor heterogeneity, making it more challenging to treat tumor cells effectively (Figure 6).

#### Palmitoylation in immune checkpoint regulators

Much attention has been paid to the research surrounding immune checkpoints and immunotherapy. The expression of PD-L1 is dependent on its palmitoylation by ZDHHC3 or ZDHHC9, which prevents its targeting to lysosomes for degradation and inhibits the function of CD8^+^ T cells, facilitating immune escape.^142^^,^^322^ However, silencing of ZDHHCs suppressed tumor progression by reversing immunosuppressive, supporting inactivating palmitoylation of key point protein to potentiate T cell immunotherapy.^323^^,^^324^ To enhance the lethality of cytotoxic T cells, a competitive peptide has been developed to inhibit PD-L1 and with surpassing specificity compared with 2-BP. These findings highlight the significant antitumor effects of targeted palmitoylation *in vitro*.

TIM-3 is expressed on various immune cells, including Th1 cells, cytotoxic lymphocytes, monocytes, macrophages, NKs, and DCs. It functions as a negative regulator of immune responses by interacting with its ligands, such as galectin-9, phosphatidylserine, CEACAM1, and HMGB1, particularly in the context of autoimmunity and cancer.^325^ In the TME, Tim-3 on tumor-infiltrating DCs binds to HMGB1, blocking the transport of nucleic acids into endosomes and suppressing innate immune responses to tumor-derived nucleic acids.^326^ Recent research indicated that TIM-3 is palmitoylated by ZDHHC9, which stabilizes TIM-3 by preventing its interaction with HRD1 from polyubiquitination degradation. This modification enhances TIM-3 expression on immune cell surfaces, contributing to immune exhaustion and dampening antitumor responses. Targeting Tim-3 palmitoylation could offer a therapeutic strategy in cancer immunotherapy.^327^

Tregs facilitate tumor immune evasion by impairing the activity of effector cells via multiple mechanisms within the TME. Tregs depend on the master transcription factor fork-head box protein p3 (Foxp3) to perfume this regulatory function. However, inhibition of ZDHHC2, ZDHHC3, and ZDHHC7, which are responsible for palmitoylating Foxp3, might reduce the nuclear localization and stability of Foxp3 in both peripheral lymphoid tissues (e.g., spleen, lymph nodes) and tumor-infiltrating Tregs. This impairment compromises the transcriptional activity of Foxp3, thereby weakening Treg-mediated immunosuppressive functions within the TME.^185^ T helper cell 17 (Th17) differentiation stimulator STAT3 can be reversible palmitoylated. ZDHHC7 is responsible for its membrane recruitment and phosphorylation, and APT2 enables translocate to the nucleus.^328^

Macrophages also known as tumor-associated macrophages (TAMs) in the TME, can either promote or oppose tumor growth, depending on different phenotypes induced by various exogenous stimuli.^329^ Enhanced lipid accumulation and metabolism are required for the differentiation and activation of TAMs, which upregulate CD36 to increase LCFA uptake, which was accompanied by intracellular lipid accumulation and higher FAO.^329^

#### Palmitoylation in adipocytes

An HFD induces FA metabolic disorders, endowing tumor cells with a survival advantage. CD36 regulates FA endocytosis through dynamic palmitoylation, which is crucial for FA uptake in adipocytes. Palmitoylated CD36 captures FAs at the cell surface, while depalmitoylation initiates the endocytic process, internalizing FAs into the cell.^53^ In the TME, increased uptake of PA by cancer cells leads to impaired CD8^+^ T cell infiltration.^330^ Sterol regulatory element-binding protein 1c (SREBP1c) stimulates the generation of multiple lipid genes, regulating lipid homeostasis through a feedback system. It has been reported that inhibiting the SREBP pathway reduces intracellular PA and HCC progression by suppressing tumor-promoting cytokines such as IL-6 and TNF-α. Palmitoylation-driven PHF2 ubiquitination and degradation reduce the destruction of SREBP1c, thereby increasing SREBP1c-dependent lipogenesis and the malignant progression of HCC.^331^

## Targeting PA and palmitoylation-associated pathways for cancer therapies

### Targeting PA metabolic pathways

Since glycolysis is connected with FA metabolism and FA acts as pro-angiogenic growth factor, reducing hyper-glycolysis by controlling FA metabolism in cancer cells promises a better strategy against tumor progression. FABPs are potential cancer therapeutic targets, as they act as angiogenic and metabolic mediators in endothelial cells and carcinoma cells. Meanwhile, FASN has arguably received the most widespread interest in terms of cancer therapies targeting dysregulated lipid metabolism. Inhibition of FASN caused a reduction in *de novo* palmitate synthesis and EGFR palmitoylation, providing an alternative approach to blocking EGFR activity.^203^ Early-generation FASN-targeted agents such as C75, orlistat, and cerulenin initially showed great promise in preclinical studies, including blocks TLR/MyD88 signaling in neutrophils and increasing the survival rate of LPS-induced septic shock models.^138^ However, they also demonstrated significant clinical hurdles, including deleterious systemic side effects characterized by drastic weight loss and anorexia.^332^ The good news is that next-generation FASN inhibitors, including TVB-3166 and TVB-2640 (Denifanstat), have shown tremendous antitumor potential in preclinical breast and CRC models, enabling patients to actually benefit from FASN inhibition.^333^

ACLY expression and activity are increased in a variety of tumor types and, considering its role in generating acetyl-CoA, ACLY inhibition could represent a well-tolerated therapeutic strategy. The relatively high drug concentrations required to completely inhibit ACLY activity and differences in ACLY activity dependency for glycolysis-fueled lipogenesis rates currently limit cancer therapeutics targeting ACLY. However, a recent study showed that the inhibitor SB-204990 can suppress tumor growth in several cancer xenograft modules.^63^ Soraphen is a highly potent inhibitor of ACC, increasing the abundance of poly-unsaturated FAs that are more susceptible to peroxidation, rendering cells more susceptible to oxidative stress-induced cell death.^334^ The ACC inhibitors TOFA and ND646 have shown significant antitumor effects.^335^

Given the important roles of lipid synthesis and desaturation in normal cellular function, the use of lipogenesis inhibitors could serve as antineoplastic agents and as chemotherapeutic sensitizers. Studies have shown that inhibition of canonical SCD-mediated desaturation induces apoptosis and growth inhibition in cancer cells through a variety of mechanisms.^41^ SREBP protects cancer cells from lipotoxicity and ER stress induction by maintaining *de novo* lipid synthesis and desaturation via SCD.^336^ Inhibition of lipid desaturation in cancer cells can lead to the loss of integrity of essential membrane structures, depletion of energy, and ER stress. However, re-expression of SCD or addition of exogenous oleic acid prevented the induction of ER stress and restored mitochondrial function, indicating that maintenance of FA desaturation is a crucial part of the SREBP pathway.^337^ Hence, for some compensatory desaturated pathways that utilize FADS2 to produce sapienate from PA, SCD, and FADS2 inhibitors combine to block any pathways for obtaining desaturated FAs.^43^

### Targeting ZDHHCs and depalmitoylation enzymes

To date, no drugs have been specifically developed to inhibit DHHC-PATs. Among the available inhibitors, 2-bromopalmitate (2-BP) is the most widely used. It irreversibly inhibits palmitoylation by occupying the lipid binding cavity of the ZDHHC proteins.^338^ Nevertheless, 2-BP also regulates lipid synthesis, transport, metabolism, and lacks specificity, as it can influence other enzymes such as APT1 and APT2. In addition to 2-BP, tunicamycin and cerulenin also have been reported as inhibitors.^339^ Recently, a novel broad-spectrum inhibitor of ZDHHCs, cyanomethyl-*N*-myracrylamide (CMA), was developed. CMA can inhibit the palmitoylation of protein substrates at a lower concentration and decrease toxicity.^340^

Furthermore, increasing evidence suggests that inhibiting depalmitoylase to alter protein function and affect disease development is a promising therapeutic approach. Recent studies have shown that inhibiting PPT1, a target for chloroquine derivatives such as hydroxychloroquine (HCQ) or GNS561, significantly boosts the antitumor effects of anti-PD-1 antibodies in melanoma treatment.^249^ This synergistic approach results in reduced tumor growth and improved survival rates in mouse models. The dimeric chloroquine compound (DC661) is a novel PPT1 suppressor that increases lysosomal membrane permeabilization, blocks autophagy, and induces cytoxicity.^250^ HCQ or DC661 suppresses PPT1, triggering the activation of the cyclic GMP-AMP synthase/stimulator of the cGAS/STING/TBK1 pathway and promoting the secretion of IFN-β in macrophages.^250^ PPT1 inhibition induced lysosomal lipid peroxidation, driving lysosomal cell death. Additionally, DC661 enhances T cell-mediated toxicity, promotes adaptive immunity, and induces tumor rejection, suggesting a rational combination of immunotherapy and lysosomal inhibition as a unique immunogenic form of cell death.^341^

Palmostatin B is a widely used tool for blocking depalmitoylase activity at the current stage. However, palmostatin B is not specific to APT1/APT2 and inactivates many other lipid-processing serine hydrolases, including ABHD17A-C, PNPLA6, and FASN.^173^ The use of palmostatin B or *APT* depletion increases CD95-mediated apoptosis.^342^ Although palmostatin B inhibits the palmitoylation of MCAM to prevent invasion of melanoma tumor cells, APT1 and APT2 inhibitors ML348 and ML349 have no effect on MAPK signaling or growth in several N-Ras-dependent melanoma cell lines.^343^ These findings indicated that different inhibitors may selectively target specific molecular characteristics of tumors or cellular components. While ML211, a dual inhibitor of APT1/APT2, has been developed, its therapeutic efficacy *in vivo* is significantly constrained by poor solubility.^344^ The characterization and mechanism of palmitoylation-related enzyme inhibitors are listed in Table 3.

**Table 3:** Characterization and mechanism of palmitoylation-related enzyme inhibitors

Enzymes	Inhibitor	Characteristic/Disease	Mechanism	Reference
ZDHHCs	2-BP(2-bromopalmitate)	widely used and irreversibly inhibits ZDHHCs	occupying the lipid binding cavity of the ZDHHC protein	Bu et al.^154^
	CMA (cyanomethyl-N-myracrylamide)	CMA reduced EGFR acylation, and inhibited downstream AKT phosphorylation	a similar DHHC labeling spectrum as 2BP	Azizi et al.^340^
	tunicamycin		directly inhibits transfer of palmitate to protein in a cell-free system	Lan et al.^339^
	cerulenin	inhibition of T24 cell proliferation	inhibited the incorporation of [^3^H]palmitate into p21 in intact T24 cells	Lan et al.^339^
PPT1	HCQ	melanoma	deacidifying the lysosome and inhibiting autophagy, induced the secretion of interferon-β in macrophages	Sharma et al.^249^
	dimeric CQ (DC661)	melanoma	increase lysosomal membrane permeabilization, inhibiting autophagy and decreasing cell viability	Rebecca et al.^250^
	CQ (Lys05)	melanoma, colon cancer, and breast cancer	similar to HCQ	Rebecca et al.^345^
	GNS561	hepatocellular carcinoma	modulation of lysosomal functions, and synergy with immune checkpoint inhibitors	Brun et al.^346^
	DAPKA	human neuroblastoma	enhanced the killing of tumor cells by chemotherapeutic drugs	Dawson et al.^347^
ABHD17	ABD957	chronic lymphocytic leukemia	targeted disruption of N-Ras depalmitoylation; synergy with MEK inhibition	Bononi et al.^348^
	HDFP	acute myeloid leukemia	inhibits many different targets, FASN, FAAH, AADACL1, MAGL	Bononi et al.^348^
APT1	Palmostatin B	NRAS mutant melanoma	loss of precise homeostatic localization of Ras proteins in cells	Vujic et al.^343^
	Palmostatin M	acute myeloid leukemia	another inhibitor structurally related to palmostatin B	Remsberg et al.^349^
	ML348	lung cancer	located above the catalytic triad and blocks it by contacting hydrophobic residues	Hernandez et al.^350^
APT2	ML349	melanoma	markedly reduced Raf and MEK activation	Hernandez et al.^350^
APT1 and APT2	ML211	*HRAS* mutant cancer	inhibit LYPLA1 and LYPLA2 depalmitoylation	Šprager et al.^344^
	Orlistat	breast cancer, HCC	Inhibition of fatty acid synthase (FASN)	Schcolnik-Cabrera et al.^76^

### Targeting palmitoylated proteins

Directly targeting palmitoylated proteins may be an effective approach in diseases where genetic mutations or palmitoylation play a determinant role, such as through competitive inhibition of substrate palmitoylation. However, there is currently limited clinical trial evidence supporting these strategies.

The TEAD transcription factors are known to undergo auto-palmitoylation, which is essential for their interaction with YAP/TAZ and subsequent activation of transcriptional activity. A new generation of TEAD inhibitors, such as VT3989, competitively binds to a conserved palmitate-binding pocket in TEAD proteins, blocking auto-palmitoylation.^351^ TEAD inhibitors combined with osimertinib have shown significant synergy in preclinical studies on EGFR mutant non-small cell lung cancer cell lines and xenograft models derived from NCI-H1975 and HCC827 cell lines.^352^ This combination therapy also decreases drug tolerance and residual disease, further supporting the potential of VT3989 to enhance the durability of osimertinib’s antitumor efficacy. Although targeting the Hippo pathway holds promise for cancer therapy, potential kidney damage remains a significant concern.

In our study, we demonstrated that specific penetrating peptides can effectively attenuate AKT palmitoylation and activation by repressing AKT modification, thereby diminishing liver tumorigenesis.^154^ Additionally, metformin can reduce FASN expression, which affects AKT palmitoylation, disrupting its membrane attachment and phosphorylation. This suppression of the FASN/AKT and MAPK pathways contributes to metformin’s anti-inflammatory effects in macrophages.^353^ Similarly, a peptide (PD1-PALM) was designed to competitively inhibit PD-1 palmitoylation and expression in the whole cell.^220^ Recent studies have reported that enhancing TIM-3 stability through palmitoylation promotes immune exhaustion and suppresses antitumor immunity. Specifically, a peptidic inhibitor for TIM-3 palmitoylation has been developed, accelerating TIM-3 degradation and enhancing antitumor immunity mediated by CAR-T cells and NK cells.^327^

It has been shown that the metastasis of CRC can be prevented by blocking the palmitoylation of Rap2b using a high-affinity peptide (PTG-101).^354^ Given the importance of PD-1 and PD-L1 in both cancer cells and immune cells, polypeptide sequences containing PD-L1 palmitoylation sites were developed to inhibit the palmitoylation of endogenous PD-L1 by competitively binding to ZDHHC3. These polypeptides effectively reduce the palmitoylation of PD-L1 and significantly decrease PD-L1 expression in tumor cells.^322^ Activating KRAS mutations are present in 25% of human cancer. Recent efforts led to the development of pharmacological inhibitors targeting the KRAS^G12C^ mutant. Therefore, under the mutations, in combination with targeting key palmitoylated protein therapy may be worth exploring.^355^

## Perspectives and challenges

The advent of advanced detection techniques has enabled the construction of a comprehensive map of the enzymes and substrates involved in palmitoylation. The biochemical mechanisms underlying reversible protein palmitoylation are increasingly understood, and significant progress has been made in recent years. However, translating these findings into targeted cancer therapies faces several challenges.

One major challenge lies in the application of dietary restriction approaches, which have been proposed and validated in preclinical studies as potential therapeutic strategies. However, due to concerns about tolerability and side effects, these dietary interventions are generally unsuitable as standalone clinical treatments and are instead considered adjunctive therapies. Given the well-established context, researchers have focused on PA, a key component of dietary fat, proposing strategies to reduce its consumption. These strategies not only aim to limit its role as a structural and energy source for tumorigenesis, particularly by affecting membrane dynamics, but also inhibit its predominant role in mediating palmitoylation. The homeostasis of PA, encompassing processes like uptake, synthesis, storage, and FAO, is crucial for maintaining its physiological functions. However, disruptions to this balance, caused by genetic, epigenetic, transcriptional, post-transcriptional, or PTM-related alterations in key regulatory proteins across different cells or tissues, can lead to various diseases such as cancer. Given the essential role of PA in cellular functions, limiting or targeting PA-associated pathways could impact normal cells and potentially induce cytotoxic effects. Consequently, inhibitors targeting enzymes such as FASN are currently under preclinical evaluation for cancer therapies, although some drugs targeting these pathways have already been approved for obesity treatment.

Palmitoylation, as one of the most predominant PTMs, plays a wide range of functional roles in cellular processes. However, identifying palmitoylated proteins, especially under dynamic and endogenous conditions, remains a significant challenge, although some methods have been reported.^356^ Current detection methods, such as the biotin-switch assay, primarily rely on *in vitro* techniques that involve adding biotin to label palmitoylated proteins. To address these limitations, there is an urgent need to develop pan or specific antibodies that can recognize palmitoylation modifications on specific proteins. Such antibodies would enable the detection of endogenous and *in situ* palmitoylation changes, providing deeper insights into its physiological and pathological roles. Due to the strong hydrophobicity of LCFAs, the instability of thioester bonds, and the ability of different lipids to modify the same protein and even the same cysteine residue through thioester bonds, the generation of palmitoylation-specific antibodies remains a significant challenge in the field, despite the potential of *in vitro* palmitoylation assays to facilitate the incorporation of this modification for generating the specific antibody.

In palmitoylation studies, a single protein can be palmitoylated by multiple ZDHHC enzymes at the same or different residues, leading to similar or distinct functional outcomes. These findings raise critical questions about the specific roles of these enzymes and modifications in downstream functions, which may vary depending on the cell or tissue context or in response to various extracellular and intracellular stimuli. To address these complexities, developing more sensitive and dynamic approaches for detecting dynamic changes in endogenous palmitoylation is essential. For instance, enriching samples using potential pan-palmitoylation antibodies under dynamic conditions, we can enhance the specificity of mass spectrometry analysis for more accurate detection of lipid modifications.^357^ On the therapeutic front, while strategies to create specific inhibitors targeting individual ZDHHC enzymes have been proposed for cancer therapy, their application is complicated by the fact that ZDHHC, similar to other enzymes or kinases, often has multiple substrates. This substrate diversity may limit the efficacy of such inhibitors. However, if a particular ZDHHC enzyme is found to activate multiple oncoproteins, targeting it could provide significant therapeutic benefits for cancer treatment. Thus, further research is needed to better understand these mechanisms, which will help us optimize strategies for effective intervention.

Different from other PTMs, palmitoylation stands out due to the hydrophobic nature of lipid-mediated modification. This characteristic often enhances the affinity of substrates for membranes, facilitating their localization to various cellular compartments such as the PM, endoplasmic reticulum, mitochondria, or Golgi apparatus. Such membrane localization is crucial for the substrates to perform their biological functions effectively. Additionally, palmitoylation is also known to interact with other PTMs, such as phosphorylation, ubiquitination, or acetylation, to create a complex regulatory network. These interactions can dynamically modulate protein stability, trafficking, and signaling pathways, ultimately influencing downstream cellular processes. Exploring this intricate interplay is essential for a deeper understanding of how palmitoylation cooperates with other modifications to fine-tune cellular function and signaling networks.

Palmitoylation plays a critical role in regulating various cellular processes, including cell death pathways such as apoptosis, necroptosis, and ferroptosis. Its involvement in these processes makes it a significant player in cancer biology, as dysregulated cell death is a hallmark of cancer. Leveraging palmitoylation’s influence on cell death offers a valuable strategy for enhancing the efficacy of cancer therapies. Combining palmitoylation modulators with cell death inducers may sensitize cancer cells to therapeutic interventions. This is particularly relevant in the context of the immunosuppressive TME, where targeting palmitoylation pathways could potentially enhance immune cell activity and reduce cancer cell survival. Thus, targeting palmitoylation or its regulatory enzymes (such as palmitoyltransferases and depalmitoylases) offers a promising avenue to disrupt tumor-supportive signaling while reprogramming the TME. Combining such strategies with immune checkpoint inhibitors or other immunotherapies may provide a synergistic effect, improving overall therapeutic outcomes. This approach holds the potential for more precise and effective cancer treatment strategies by simultaneously addressing cancer cell survival and immune evasion mechanisms.

With a deeper understanding of palmitoylation homeostasis, its regulation of accumulating proteins, and its roles in diverse cellular functions, immune responses, and the TME, significant progress is being made in unraveling its biological and pathological implications. Despite the substantial challenges in studying palmitoylation, such as its dynamic and reversible nature, and in developing specific and efficient therapeutic strategies targeting these pathways, the field holds immense potential. Meanwhile, decoding the dynamic alterations in palmitoylation and related pathways will not only enhance our understanding of complex diseases like cancer but also identify novel therapeutic targets. This knowledge could lead to innovative cancer treatment approaches, including the development of drugs targeting palmitoylation machinery or pathways. Moreover, integrating such therapies with immune-based treatments, such as immune checkpoint inhibitors, could provide synergistic effects, effectively targeting both tumor growth and immune evasion. As research progresses, these insights are expected to pave the way for more precise and personalized therapeutic strategies, ultimately improving outcomes for patients with cancers and other diseases influenced by palmitoylation.

## Resource availability

### Materials availability

This study did not generate new unique materials or reagents.

### Data and code availability

No new data were created or analyzed in this study.

## Funding and acknowledgments

The authors sincerely apologize to all those colleagues whose important work was not cited in the paper owing to space limitations. We thank members of the Guo laboratory for critical reading and kind suggestion of the manuscript. This work was supported in part by the 10.13039/501100012166National Key Research and Development Program of China (2023YFC3402100 to J.G.), the 10.13039/501100001809National Nature Science Foundation of China (82473404 to W.X.), and the 10.13039/501100003453Natural Science Foundation of Guangdong Province (2022A1515220004 to J.G., 2024A1515010945 to W.X., and 2023A1515010384 to Q.J.). The figures were generated with Biorender.com.

## Author contributions

P.S. and J.G. conceived and wrote this review together with the help of Q.J., W.W., X.X., X.W., L.B., and W.X. All authors contributed to the manuscript and approved the final version.

## Declaration of interests

W.W. is a co-founder and consultant for the ReKindle Therapeutics.
